# Assessing surface water quality in Hungary’s Danube basin using geochemical modeling, multivariate analysis, irrigation indices, and Monte Carlo simulation

**DOI:** 10.1038/s41598-024-69312-8

**Published:** 2024-08-11

**Authors:** Omar Saeed, András Székács, Győző Jordán, Mária Mörtl, Mostafa R. Abukhadra, Ahmed M. El-Sherbeeny, Péter Szűcs, Mohamed Hamdy Eid

**Affiliations:** 1 Doctoral School of Environmental Science, Hungarian University of Agriculture and Life Sciences (MATE), Páter Károly u. 1, Gödöllő, 2100 Hungary; 2 Agro-Environmental Research Centre, Institute of Environmental Sciences, Hungarian University of Agriculture and Life Sciences, Páter Károly u. 1, Gödöllő, 2100 Hungary; 3 Eötvös Loránd University (ELTE), Budapest, Hungary; 4 Geology Department, Faculty of Science, Beni-Suef University, Beni-Suef, 65211 Egypt; 5 Industrial Engineering Department, College of Engineering, King Saud University, P.O. Box 800, 11421 Riyadh, Saudi Arabia; 6 Institute of Environmental Management, Faculty of Earth Science, University of Miskolc, Miskolc, 3515 Hungary

**Keywords:** Hydrochemical assessment, Irrigation and drinking WQIs, PC analysis, Machine learning models, Danube River, Environmental sciences, Hydrology

## Abstract

Evaluation of water quality is crucial for managing surface water effectively, ensuring its suitability for human use, and sustaining the environment. In the lower Danube River basin, various methods were employed to assess surface water quality for irrigation, drinking, human health risk purposes and the main mechanism control the surface water chemistry. These methods included water quality indicators (WQIs), complex statistical analyses, geographic information systems (GIS), Monte Carlo simulation, and geochemical modeling. Physicochemical analyses of surface water samples revealed primarily Ca–Mg–HCO_3_^−^ is the dominant water types. Principal component analysis (PCA), ionic ratios and piper, chloro alkaline index, Chadha, and Gibbs diagrams identified three distinct water characteristics influenced by water-rocks interaction, evaporation, ions exchange, and human activities. The geochemical modeling showed Danube River water’s strong ability to dissolve gypsum, halite, and anhydrite (SI < 0) and precipitate aragonite, dolomite, and calcite with saturation index (SI) value greater than 0 along its flow path. The irrigation water quality index (IWQI = 99.6–107.6), sodium adsorption ratio (SAR = 0.37–0.68), sodium percentage (Na% = 13.7–18.7), soluble sodium percentage (SSP = 12.5–17.5), Potential Salinity (PS = 0.73–1.6), and Residual Sodium Carbonate (RSC = − 1.27–0.58) values were used, mainly indicating acceptable quality with some limitations. Danube River water was unsuitable for drinking based on WQI value (WQI = 81–104). Oral exposure of children to specific components showed a higher hazard index (HI > 1) compared to adults, indicating a 2.1 times higher overall non-carcinogenic risk hazard index. However, Monte Carlo simulation demonstrated negligible iron, manganese, and nitrate health hazards for both age groups. These findings are valuable for water quality management decisions, contributing to long-term resource sustainability.

## Introduction

Water constitutes a crucial and invaluable natural asset integral to the environmental system^1–3^. The global challenge of inadequate access to clean and safe drinking water affects over a billion individuals^4,5^. Tragically, an annual toll of 6–8 million lives is attributed to water-related illnesses and natural disasters, underscoring the profound significance of addressing water supply issues on a global scale^6,7^. Surface water, among the freshwater sources, assumes paramount importance for various purposes, including residential, recreational, agricultural, and commercial applications^8,9^. The endpoint for used water, commonly referred to as wastewater, manifests within the aquatic milieu, encompassing rivers, ponds, or other aqueous reservoirs^10–13^. Among these water resources, rivers play a pivotal role as a supply of water for individuals, crop production, and industrial processes. The increasing pollution of surface water due to human activities and the deposition of pollutants from the atmosphere, such as heavy metals, is a pressing concern, especially in urban regions, highlighting the urgent need for comprehensive environmental management strategies.

Surface water quality is adversely affected by various factors such as human-induced effects, floodplain chemical constituents, geochemical parameters, and the interaction between natural water and lithogenic origins^13–15^. These elements collectively contribute to the degradation of surface water quality, presenting significant risks to both ecological systems and human health^1,13^. Surface water quality is influenced by a spectrum of contaminants, encompassing inorganic, organic, and biological agents, Including both non-toxic and highly toxic heavy metals^10,16^, biodegradable materials such as refuse, fecal matter, and sewage^17,18^. Both natural phenomena and anthropogenic actions, such as the discharge of industrial effluents, agricultural runoff, and residential sewage into river systems, exacerbate the degradation of water quality^13,19–21^. Hence, performing an initial evaluation of these environmental assets is imperative to guaranteeing their sustained conservation over the long term^22^. Moreover, consistent monitoring of surface water quality and quantity is vital for promoting environmental well-being and realizing sustainable development objectives, exemplified by specific goals like “Aim 6: water purification and sanitation” and “Aim 14: Life beneath water” within the framework of the Sustainable Development Goals (SDGs)^23,24^. Executives, policymakers, and governments employ the Water Quality Index (WQI) as a tool to assess the present condition of water quality. This index integrates measurable parameters and presents outcomes through a numerical rating system, classifying water quality on a spectrum from good to bad^25^. Water quality is determined using a comprehensive method that takes into account all of the physical, chemical, and biological qualities of the water^13^. Given the heightened susceptibility of surface water supplies to harmful constituents, particularly in developing nations, assessing freshwater quality becomes imperative^26,27^. The widespread adoption of the Water Quality Index (WQI) technique by numerous countries attests to its singular value and ease of comprehension for evaluating the overall condition of rivers^13,28^. Alterations in water quality can have various repercussions on irrigation, leading to a decline in the productivity and fertility of agricultural soil. Excess salts, for instance, can adversely affect the soil’s structure, texture, permeability, and aeration^29^. Improper irrigation techniques utilizing polluted surface water, as well as soil mismanagement, may result in the formation of too many soluble salts, causing the soil to become alkaline^30^. Consequently, assessing the water’s quality for agricultural purposes is crucial, especially in dry and semiarid regions or countries where salt and sod development often occur in agricultural soil^13^. Water management for irrigation and field distribution considers factors such as corrosion and scale potentials, addressing undesirable changes in water quality that may result in hydrological, economic, and aesthetic losses^31^. Excessive contamination can potentially diminish efficiency and lead to tube obstruction in industrial equipment, thereby escalating the overall costs of industrial operations^29,32^. In the broader context encompassing drinking, irrigation, and commercial uses, water quality emerges as a pivotal concern in water supply management, necessitating thoughtful planning for the judicious utilization of surface water^33^. In this investigation, we employed Monte Carlo methodology to assess the non-carcinogenic risk connected with exposure to Iron, Manganese, and Nitrate within the lower Danube River basin. Monte Carlo simulation offers a powerful tool for quantifying uncertainty and variability inherent in environmental risk assessments, particularly when dealing with complex systems and multiple contaminants. Our investigation contributes to a more thorough knowledge of the health implications posed by these contaminants, facilitating the development of targeted mitigation strategies to safeguard human health and ecosystem integrity in this vital riverine ecosystem. The Danube River, Europe’s second-longest river, rises in Germany’s Black Forest Highlands and flows over 2800 km. Before entering the Black Sea, it flows through nine countries, including Germany, Slovakia, Austria, Hungary, Croatia, Serbia, Ukraine, Bulgaria, and Romania. Its catchment area encompasses around 817,000 km^2^^34^, the Danube River boasts a diverse aquatic ecosystem inhabited by a rich variety of plants and animals. However, this ecosystem is not immune to the potential threat of heavy metal pollution. Accumulation or increasing of PTEs in aquatic environments can impact various components of aquatic life, encompassing fish (ichthyofauna), benthic fauna residing on the riverbed, and macrophytes, aquatic plants^35,36^.

This study represents the inaugural comprehensive endeavor to assess temporal and regional changes in river water’s physicochemical qualities. It investigates the causes and factors causing spatiotemporal discrepancies in the water purity of the Danube River using statistical approaches, employing multivariate statistical methods. The investigation also scrutinizes the suitability of the water’s viability for various purposes, including irrigation, drinking, and health risks with Monte Carlo simulation. The outcomes of this study are anticipated to furnish policymakers with valuable insights for advancing the UN Goals-2030 and achieving Sustainable Development Goals (SDGs2030) within this ecosystem. The focus lies on mitigating pollution and restoring the ecological balance of the riverine environment.

## Materials and methods

### Study geographic features and water sampling

**Figure 1:**
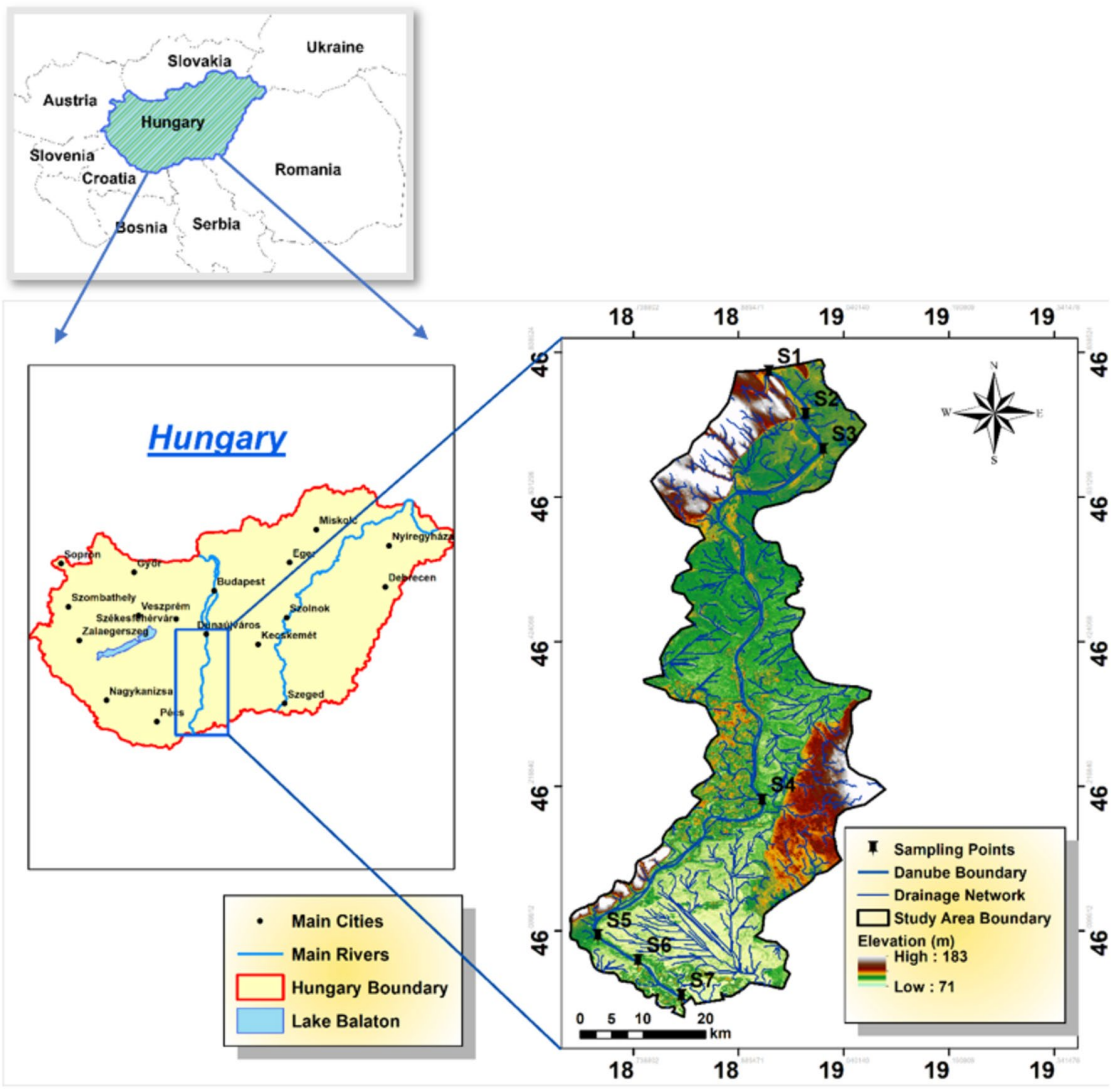
Study area and the distribution of sampling points in the lower Danube River basin.

The study focused on evaluating Hungary’s Danube River water quality, specifically within Baja, Dunaföldvár, and Hercegszántó, spanning January to December 2019. Sampling occurred at seven monitoring locations throughout southern Hungary’s Danube River, between coordinates 46°10′54.4548″ N and 18° 57′ 15.5016″E. These samplings were identified as “S1, S2, S3” for the right bank, left bank, and mid-streams of samplings in Dunaföldvár, “S4” for the left bank in Baja, and “S5, S6, S7” for the right bank, left bank, and mid-streams in Hercegszántó and their distribution map was created using QGIS 3.34.3. (Fig. 1). Samplings intended to encompass varied land use activities, such as agricultural, commercial, and residential areas on both riverbanks, potentially contributing to water contamination. Collected samples offer a comprehensive insight into water quality dynamics in these areas, which is crucial for assessing anthropogenic impacts on the Danube River. Beyond the studied cities, the Danube River then flows through additional countries before reaching the Black Sea. Hungary experiences a continental climate with hot, low-humidity summers and cold, snowy winters. Moderate precipitation occurs throughout the year, resulting in significant regional weather variations. Climatological data from 1991 to 2020 indicates an average maximum temperature of 29 °C in July–August and an average minimum temperature of − 2.59 °C in December–February. Substantial rainfall, averaging 619 mm annually, typically falls between May and July in the study area.

### Samples collection and analytical approaches

In 2019, an overall of 85 water samples were methodically gathered from seven predetermined sampling locations (Fig. 1) along a 120-km segment of the Danube River, adhering to the upstream-to-downstream flow. The seven locations covered all different landcover around Danube River including agricultural land, industrial sector, urban area, and small streams from other cities that discharge its water to the river and could affect its water quality regarding potential toxic elements. The measured parameters included the most important indicators or elements (15 parameters) that could explain the water quality concerning drinking, irrigation, and health risk issues, as well as the mechanism governing the chemical characteristics of the river. The evaluation based on these criteria could be crucial for water management plans and decision-makers for sustainable development. To guarantee uniformity, composite samples were manually drawn from 30 cm beneath the water surface, particularly in regions with swift water currents, ensuring comprehensive representation from each sampling point. Each sample was meticulously curated by amalgamating water collected at each site on three separate occasions, yielding 10 sets of composite water samples. Before collection of samples, 500-ml polyethylene bottles were rigorously washed with chemicals, thoroughly rinsed with filtered water, and submerged in a 10% HNO3 solution overnight. The obtained samples were then sent to a laboratory with a regulated temperature of 4 °C for further chemical analysis. On-site measurements of pH, temperature (T), total dissolved solids (TDS), and electrical conductivity (EC) were conducted using specialized instruments: a pH meter and a digital thermometer (Hannah, Woonsocket, RI, USA). Additionally, TDS and EC were analyzed utilizing digital TDS and EC meters (HM digital, Redondo Beach, CA, USA). To ensure accuracy, all digital meters underwent standardization with deionized water and buffer solutions before the commencement of sample analysis. For cations analysis, the samples underwent filtration through 0.45 µm filters. Subsequently, ten drops of ultra-pure HNO3 were added to one set of samples^37^. Calcium (Ca^2+^) and magnesium (Mg^2+^) contents were assessed using the EDTA titrimetric method, which employs ethylenediaminetetraacetic acid. Sodium (Na^+^) and potassium (K^+^) ion contents were measured utilizing a flame photometer (ELEX 6361, Eppendorf AG, Hamburg, Germany). Total hardness (TH) was evaluated using Eriochrome Black-T (C_20_H_12_N_3_O_7_SNa) and ammonium chloride (NH4Cl) indicators in an EDTA solution. To assess chloride (Cl^−^) concentrations, a titration method employing silver nitrate (AgNO_3_) and potassium chromate (K_2_CrO_4_) indicators was employed. For the detection of bicarbonate (HCO_3_^−^) and carbonate (CO_3_^2−^) concentrations, a titrimetric technique involving a standard solution of sulfuric acid (H_2_SO_4_) and methyl orange indicator was utilized. Additionally, Cl^−^ concentrations were determined through titration with silver nitrate. Concentrations of sulfate (SO_4_^2−^) and nitrate (NO_3_^−^) were measured using a spectrophotometer based on the visible ultraviolet (UV) spectrum (DR/2040- Loveland, CO, USA). Iron and manganese were assessed through flame atomic absorption spectrometry (FAAS).

### Quality assurance and control

The water quality analysis followed the standard methodology specified by the American Public Health Association (APHA) in 2012^38^. To ensure the accuracy of on-site testing equipment, we carefully standardized all instruments with deionized water and buffer solutions before starting sample analysis. Various quality assurance procedures were applied during the water sample examination. The analytical processes were validated by instrument calibration, accuracy, and predictability evaluations. Charging balance errors (CBE) were evaluated following field observations and then validated in the laboratory. The samples were examined in triplicate, and the average values were also given. Equation (1) ^39^ was used to analyze anion-cation balance errors based on the principle of neutrality, which states that the sum of the number of cations equals the sum of the number of anions in meq/L − 1. The CBE for all examined samples was within the permissible range of ± 5%.

1 $${\text{CBE}} = \frac{{\Sigma {\text{Cations}} - \Sigma {\text{Anions}}}}{{\Sigma {\text{Cations}} + \Sigma {\text{Anions}}}}*100$$

Furthermore, the quality assurance of the analytical procedure was doublE−checked through a meticulous examination involving Certified Reference Material (CRM) and the blank technique analysis.

### Indexing techniques

#### The index of processes influencing surface−water chemistry (IPIC)

An alternative method for discerning the provenance and mutual dependencies of principal constituents involves the juxtaposition of [SO_4_^2−^] against [Ca^2+^], [HCO_3_^−^ + SO_4_^2−^] against [Ca^2+^ + Mg^2+^], [Na^+^] against [Cl^−^], and [HCO_3_^−^] against [Ca^2+^ + Mg^2+^]. In this investigation, the chloro-alkaline indices.

(CAI-I and CAI-II) (Eqs. 2 and 3) were employed to ascertain the mineral composition within the aquifers and the ionic exchanges occurring in surfacE−water systems^39^.

2 $${\text{CAI}} - {\text{I}} = \frac{{{\text{Cl}}^{ - } - \left( {{\text{Na}}^{ + } - {\text{Ca}}^{2 + } } \right)}}{{{\text{Cl}}^{ - } }}$$

3 $${\text{CAI}} - {\text{II}} = \frac{{{\text{Cl}}^{ - } - \left( {{\text{Na}}^{ + } - {\text{Ca}}^{2 + } } \right)}}{{{\text{So}}_{4}^{2 - } + {\text{HCO}}_{3}^{ - } + {\text{CO}}_{3}^{2 - } + {\text{NO}}_{3}^{2 - } }}$$

#### Saturation indices (SI)

A speciation approach was applied to ascertain minerals’ saturation index (SI) in surface water (SW) samples collected throughout Hungary’s Danube River. The SI of a mineral denotes its saturation status concerning the prevailing conditions of the surrounding system. Employed within this study, the SI served as a predictive tool to assess the potential existence of responsive minerals in water, utilizing water samples rather than solid-phase samples or thorough mineral characterization^40^. Equation (4) was utilized for SI computation:

4 $${\text{SI}} = {\log}\left( {{\text{IAP}}/{\text{Ksp}}} \right)$$

IAP stands for “ion activity product,” and ${K}_{sp}$ stands for “solubility product” at a given temperature. A SI of zero implies saturation, a positive number shows oversaturation and a negative number indicates undersaturation regarding mineral equilibrium.

### Drinking water quality index calculations (DWQI)

The Drinking Water Quality Index (DWQI) is a valuable tool for assessing the overall quality of surface water, specifically in the context of its suitability for consumption^13,41^. The preliminary assessment of water quality, facilitated by the WQI, is crucial in aiding decision-makers as they navigate policy implications and plan for future water resource management. WQI, as a grading system, provides a holistic representation of the cumulative impact of diverse water quality parameters on the overall water quality^42,43^. Unlike traditional approaches that focus on absolute assessments of contamination or specific water quality parameters, WQI introduces a novel technique for evaluating the overall health of rivers^44^. The uniqueness of WQI lies in its capacity to amalgamate various environmental parameters, effectively condensing them into a singular numerical value that reflects the prevailing status of water quality. This departure from conventional methods underscores the transformative potential of WQI in providing a more comprehensive and integrative perspective on water quality assessment. The DWQI method emerges as a highly effective approach for accurately evaluating and controlling water quality, providing knowledge concerning the overall quality^45^. In Hungary’s lower Danube River basin, the calculation of WQI took into account a spectrum of critical parameters, including pH, temperature, electrical conductivity (EC), dissolved solids (TDS), dissolved oxygen (DO), sodium (Na^+^), calcium (Ca^2+^), potassium (K^+^), magnesium (Mg^2+^), chloride (Cl^−^), sulfate (SO_4_^2−^), nitrate (NO_3_^−^), and bicarbonate (HCO_3_^−^). This inclusive consideration of various indicators underscores the WQI’s ability to encapsulate a broad array of water quality factors, facilitating a more holistic and integrated assessment approach (Eq. 5).

5 $${\text{DWQI}} = \left( {\frac{{{\text{w}}_{{\text{i}}} }}{{\mathop \sum \nolimits_{{{\text{i}} = 1}}^{{\text{n}}} {\text{w}}_{{\text{i}}} }}} \right) \times \left( {100 \times \frac{{{\text{C}}_{{\text{i}}} }}{{{\text{S}}_{{\text{i}}} }}} \right)$$

The variable “w_*i*_” represents the weight assigned to each parameter, while "n" denotes the overall number of variables used in this research.

“*C*_*i*_” signifies the concentration of each chemical parameter measured in milligrams per liter for every water sample, and ‘s_*i*_” represents the corresponding World Health Organization (WHO) water quality guidelines.

The calculation of “w_*i*_” for each parameter is determined in accordance with the prescribed standards^46^ Eq. (6):

6 $${\text{w}}_{{\text{i}}} = {\text{K}}/{\text{S}}_{{\text{i}}}$$

Here, the constant of proportionality, denoted as “K,” is computed using Eq. (7):

7 $${\text{K}} = 1/\sum 1/{\text{S}}_{{\text{i}}}$$

Drinking Water Quality Index calculation (DWQI) involves assigning a weight (w_*i*_) to each surface water variable, followed by the determination of the relative weight (W_*i*_). Consequently, W_*i*_ scores were allocated for all physicochemical parameters listed in Table 1, with w_*i*_ computed using Eq. (6). The resulting scores for guidelines, unit weights (w_*i*_), and arithmetic weights (W_*i*_) for the water elements are presented in Table 1. WQI is classified into five categories Table 1S.

**Table 1:** Determining the Drinking Water Quality Index (DWQI) using the arithmetic weight method for physicochemical parameters.

Nos.	Parameters	Unit	WHO (2017) (Si)(^1^)	(1/Si)	Wi = (K/Si)	Qi = (Ci/Si) *100	WiQi
1	pH		8.5	1.176E−01	8.640E−03	9.581E+01	8.278E−01
2	EC	µs/cm	1500	6.667E−04	4.896E−05	3.119E+01	1.527E−03
3	TH	mg/L	500	2.000E−03	1.469E−04	2.276E+01	3.343E−03
4	TDS	mg/L	500	2.000E−03	1.469E−04	5.450E+01	8.005E−03
5	Ca^2+^	mg/L	75	1.333E−02	9.792E−04	7.260E+01	7.109E−02
6	Mg^2+^	mg/L	50	2.000E−02	1.469E−03	3.227E+01	4.739E−02
7	Na^+^	mg/L	200	5.000E−03	3.672E−04	8.279E+00	3.040E−03
8	K^+^	mg/L	12	8.333E−02	6.120E−03	2.153E+01	1.318E−01
9	Cl^−^	mg/L	250	4.000E−03	2.938E−04	9.800E+00	2.879E−03
10	NO_3_^−^	mg/L	50	2.000E−02	1.469E−03	1.673E+01	2.458E−02
11	HCO_3_^−^	mg/L	120	8.333E−03	6.120E−04	1.628E+02	9.966E−02
12	CO_3_^2−^	mg/L	350	2.857E−03	2.098E−04	2.619E+00	5.496E−04
13	SO_4_^2−^	mg/L	250	4.000E−03	2.938E−04	1.490E+01	4.377E−03
14	Fe^3+^	mg/L	0.3	3.333E+ 00	2.448E−01	2.000E+02	4.896E+01
15	Mn^2+^	mg/L	0.05	2.000E+ 01	7.344E−01	7.000E+01	5.141E+01
∑					1		1.016E+02

### Irrigation water quality indices

Water quality indices (WQI) are a set of physicochemical factors employed to measure water quality, reducing a massive quantity of data to a short and straightforward expression^47,48^. The irrigation water quality index (IWQI) is a powerful and informative tool for assessing and controlling soil assets and agricultural productivity. Indeed, irrigation water may complicate soil properties and crop yields owing to the numerous influences of water quality factors. The IWQI, Na%, RSC, PS, SSP, and SAR were computed using the surface−water samples’ physicochemical properties, as given in Table 2.

**Table 2:** The IWQIs, formula, and reference.

IWQIs	Formula
IWQI	${\text{IWQI}} = \mathop \sum \limits_{{{\text{i}} = 1}}^{{\text{n}}} {\text{Q}}_{{\text{i}}} \times {\text{W}}_{{\text{i}}}$
SAR	${\text{SAR}} = { }\left( {\frac{{{\text{Na}}^{ + } }}{{\sqrt {\left( {{\text{Ca}}^{2 + } + {\text{Mg}}^{2 + } } \right)/2} }}} \right) \times 100$
Na %	${\text{Na\% }} = { }\frac{{\left( {{\text{Na}}^{ + } + {\text{K}}^{ + } } \right)}}{{\left( {{\text{Ca}}^{2 + } + {\text{Mg}}^{2 + } } \right) + \left( {{\text{Na}}^{ + } + {\text{K}}^{ + } } \right)}} \times 100$
SSP	${\text{SSP}} = { }\frac{{{\text{Na}}^{ + } }}{{{\text{Ca}}^{2 + } + {\text{Mg}}^{2 + } + {\text{Na}}^{ + } }} \times 100$
PS	${\text{PS}} = {\text{ Cl}}^{ - } + { }\left( {\frac{{{\text{SO}}_{4}^{2 - } }}{2}} \right)$
RSC	${\text{RSC}} = { }\left( {{\text{HCO}}_{3}^{ - } + {\text{CO}}_{3}^{ - } } \right) - \left( {{\text{Ca}}^{2 + } + {\text{Mg}}^{2 + } } \right)$

*IWQIs are determined in meq/L.

#### Irrigation water quality index

To compute the IWQI, the following Eqs. (8–10) applied a non-dimensional scale with an interval of 0–100 to the relationship between variables such as SAR, EC, Cl^−^, Na^+^, and HCO_3_^−^^49^.

8 $${\text{IWQI}} = { }\mathop \sum \limits_{i = 1}^{{\text{n}}} {\text{Q}}_{i} \times {\text{W}}_{i}$$

Particularly, Q_*i*_ reflects the output of the quality measurement within the acceptability boundaries, and W_*i*_ represents the weight of each variable (Table 3).

9 $${\text{Q}}_{i} = {\text{Q}}_{{{\max}}} - \left( {\left( {\left( {{\text{X}}_{{{\text{ij}}}} - {\text{X}}_{{{\text{inf}}}} } \right) \times {\text{Q}}_{{{\text{imap}}}} } \right)/{\text{X}}_{{{\text{amp}}}} } \right)$$

where X_*inf*_ is the integer equivalent to the class’s lower threshold, and X_*ij*_ is the recorded number for every variable. Q_*imap*_: The class amplitude, X_*amp*_: The amplitude class to which the variable corresponds. Subsequently the W_*i*_ values were then determined using the equation below:

10 $${\text{W}}_{i} = \frac{{\mathop \sum \nolimits_{{{\text{j}} = 1}}^{{\text{k}}} {\text{F}}_{{\text{j}}} {\text{A}}_{{{\text{ij}}}} }}{{\mathop \sum \nolimits_{{{\text{j}} = 1}}^{{\text{k}}} \mathop \sum \nolimits_{{{\text{i}} = 1}}^{{\text{n}}} {\text{F}}_{{\text{j}}} {\text{A}}_{{{\text{ij}}}} }}$$

where _*i*_ is the total amount of physicochemical variables considered by the approach, varying from 1 to n; j is the number of variables considered by the approach, ranging from 1 to k; and F is the default number of element 1, A = The substantially limited of variable i by component j.

**Table 3:** The range of limit values of the parameters used in the computation of quality measurement (Qi).

Q_i_	SAR	EC (µs/cm)	HCO_3_^−^ (meq/L)	Na^+^ (meq/L)	Cl^−^ (meq/L)
0–35	≥ 12	EC < 200 or EC ≥ 3000	HCO_3_ < 1 or HCO3 ≥ 8.5	Na < 2 or SAR ≥ 9	Cl < 1 or Cl ≥ 10
35–60	6 ≤ SAR < 12	1500 ≤ EC < 3000	4.5 ≤ HCO_3_ < 8.5	6 ≤ Na < 9	7 ≤ Cl < 10
60–85	3 ≤ SAR < 6	750 ≤ EC < 1500	1.5 ≤ HCO_3_ < 4.5	3 ≤ Na < 6	4 ≤ Cl < 7
85–100	2 ≤ SAR < 3	200 ≤ EC < 750	1 ≤ HCO_3_ < 1.5	2 ≤ Na < 3	1 ≤ Cl < 4

### Utilizations of multivariate statistical approaches and GIS tools

Researchers may undertake multivariate investigations utilizing physicochemical data as well as chemical compounds (major anions and cations) to acquire a better knowledge of the surface water system and its chemistry^50^. This research used Principal Component Analysis (PCA) and Hierarchical Cluster Analysis (HCA) implemented by IBM® SPSS® Statistics 29, while Monte Carlo method was simulated using Python. To demonstrate regional and temporal fluctuations in water quality within Hungary’s Danube River distribution system, inverse distance weighting techniques (IDW) interpolation was used using QGIS 3.34.3. IDW, a deterministic technique, is used to spatially interpolate data and estimate values between observations. This approach was chosen over spline and kriging methods because of its reduced computational and modeling needs, while kriging requires more user input^51–53^. GIS mapping was employed to illustrate the geographical and temporal distribution patterns of physicochemical variables, the Drinking Water Quality Index (DWQI), main ion concentrations, irrigation water quality parameters, and statistical analysis in surface water.

### Human health risk

#### Non-carcinogenic technique for assessing human health risks

Consuming drinking water polluted with hazardous metals doubles the risk of both non-carcinogenic and carcinogenic disorders in people^36,54,55^. In this research, the evaluation of non-carcinogenic hazards associated with components such as Mn^2+^, Fe^3+^, and NO_3_^−^ was carried out utilizing procedures defined by the US Environmental Protection Agency (USEPA)^56^. The health risk evaluation methodology developed by the USEPA in 2004^36^ was used to analyze non-cancer human health hazards caused by heavy metal ions found in water bodies. This evaluation included possible dangers from eating, inhalation, and skin contact. The major hazard is from direct water use^36,56,58^. This technique uses the chronic daily intake (CDI) method to calculate the quantity of pollutants consumed by people. The CDI technique estimates the daily dosage of contaminants in kilograms Eq. (11) ^57,59^.

11 $${\text{CDI}}_{\text{oral}}=\frac{{\text{C}}_{\text{con}}\times \text{IR}\times \text{ED}}{\text{BW}\times {\text{AT}}_{nc}} \times \text{EF}$$

The attributes used in the calculations include (chronic daily intake (CDI), mg/kg/day), ${\text{C}}_{\text{con}}$ (heavy metal’s concentration, mg/L), IR (intake rate with values for adults at 2.2 L day^−1^ and children at 1.8 L day^−1^), ED (exposure duration with values for adults at 70 years and children at 6 years), BW (body weight with values for adults at 70 kg and children at 15 kg), EF (exposure frequency for both adults and children at 350 days/year), and ${\text{AT}}_{nc}$ (average time for assessing non carcinogenic risks with values for adults at 25,550 days and children at 2190 days)^56,60,61^.

The HQ (hazard quotient) is then calculated by dividing the (CDI) by the reference dose (RFD) for oral exposure (Eq. 12), respectively. The RFD oral for manganes, iron and nitrates are 0.024, 0.7 and 1.6 (mg/kg/day), respectively^36,61^.

12 $${\text{HQ}}_{{{\text{oral}}}} = \frac{{{\text{CDI}}_{{{\text{oral}}}} }}{{{\text{RfD}}_{{{\text{oral}}}} }}$$

Ultimately, the comprehensive non-carcinogenic hazards were evaluated by computing the hazard index (HI) according to Eq. (13) ^36,62^.

13 $${\text{HI}}\left( {{\text{oral}}} \right) = \sum {\text{HQ}}_{{{\text{oral}}}}$$

Toxic metals exhibiting a (HQ) or (HI) surpassing 1 are indicative of potential deleterious effects on human health, whereas those with an HI or HQ below 1, are regarded as unlikely to cause adverse consequences^36,56^.

### Monte Carlo simulation model (MCS)

**Figure 2:**
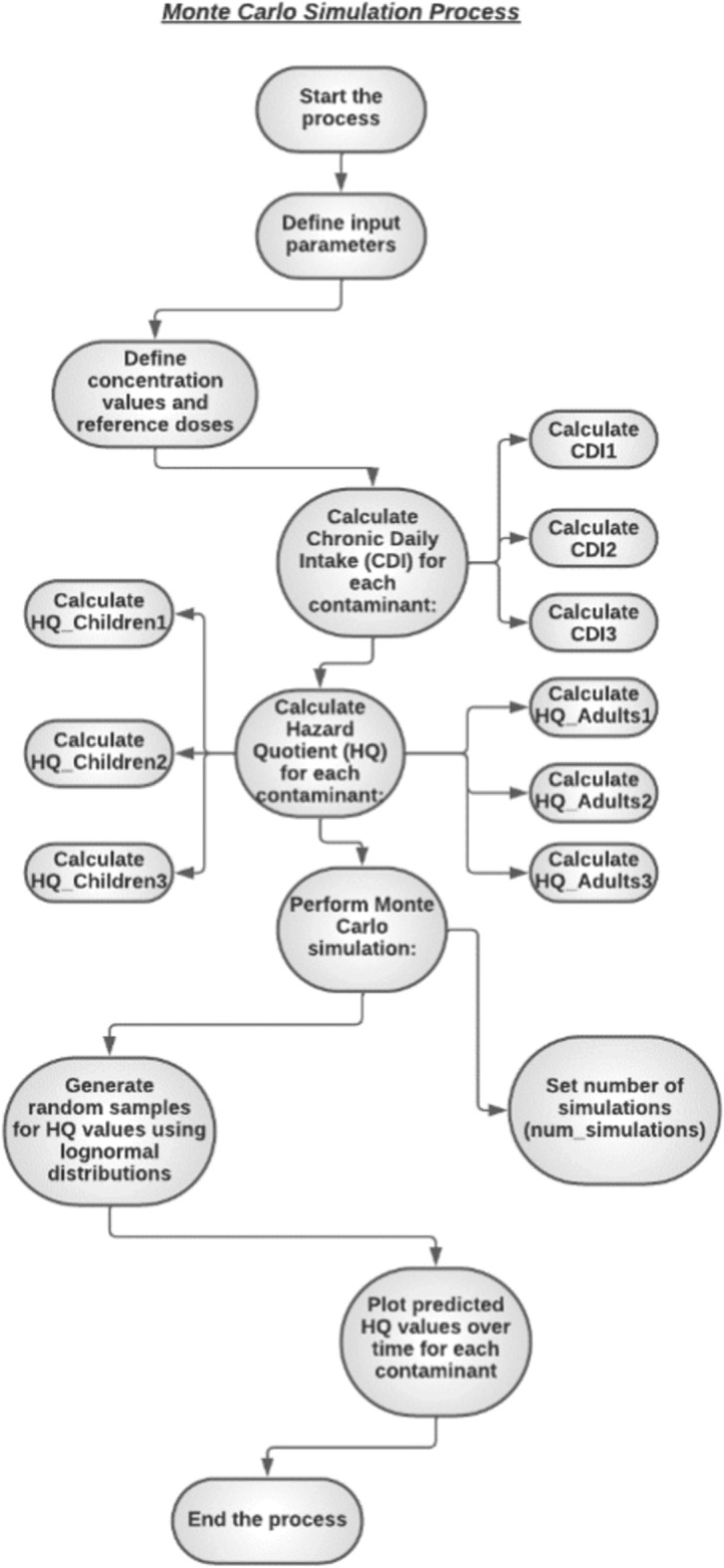
The procedural steps of a Monte Carlo simulation model.

The MCS main objective in our research was to evaluate the probabilistic distributions of several variables, including Fe^3+^, Mn^2+^, and NO_3_^−^ concentrations, ingestion rate, exposure time, average time, exposure duration, exposure frequency, body weight, skin-surface area, and permeability coefficient (Fig. 2).This analytical process was undertaken to delineate the probabilistic distributions of uncertainties associated with assessment metrics^55^.

The integration of Monte Carlo model combined with the USEPA’s health risk evaluation approach facilitates the comprehensive evaluation of uncertainties related to human health hazards from heavy metal exposure. This is accomplished by constructing a probabilistic distribution for cancer risk (CR) values. Our study used the Monte Carlo approach to predict (HQ) oral readings for adults and children. This technique reduces uncertainty and improves the reliability of non-carcinogenic health risk evaluations for iron, manganese, and nitrates in Hungary’s lower Danube River basin. The input parameters included Fe^3+^, Mn^2+^, and NO_3_^−^ concentrations, as well as the appropriate variables from Eqs. (11) and (12). To ensure the Monte Carlo simulation’s accuracy, the Python programming ran 10,000 iterations, with observed and anticipated identical HQ values, proving the model’s success. While heavy metal and NO_3_^−^ concentration distributions were determined from existing 2019 evaluated data, other factors like as exposure time, ingestion rate, skin surface area, and body weight were modeled as normal distributions to better represent their genuine distributions^36,55^.

### Limitation of research methodology

The study on the Danube River’s water quality is limited by its spatial coverage, as the 120-km segment may not represent the entire river. However, the sampling locations cover the most locations that could be affected by contaminations from anthropogenic and natural activities. Though crucial, the choice of 15 parameters might miss out on indicators, and the distribution of different types of land cover at sampling sites may not be evenly represented, potentially causing biased outcomes. Furthermore, the study may not fully consider all human-made and natural impacts on water quality, and variations in water flow dynamics and sources of pollution could impact the results more. Further research could involve more parameters, such as PTEs and stable isotopes, to detect the source of contamination and its health risk effect. Collecting samples from the groundwater with surface water in future work could explain the impact of the surface water and groundwater interaction on the water quality. The research concentrating on the lower Danube River basin in Hungary may restrict the applicability of the results to areas with varying environmental and demographic characteristics. The upper basin could be covered in future work. Additionally, although the Monte Carlo method helps mitigate uncertainty, its effectiveness is contingent upon the assumptions within the model; any errors in these assumptions could affect the credibility of health risk assessments.

## Results and discussion

### Hydrochemical properties of Hungary’s Danube river

To evaluate the surface water quality of Hungary’s Danube River, it is helpful to categorize the examined samplings according to their physicochemical properties and ion compositions, as evaluated against established standards for irrigation water activities (Table 4)^63^. The pH values of sampling vary from 7.6 to 8.6, with an average of 8.1, suggesting a slightly alkaline character commensurate with the suggested pH range (6.5–8.4) for irrigation purposes^63,64^. The EC values changed between samples significantly from 360 to 680 μS cm^−1^. However, none of the recorded EC values surpass the established irrigation standard limit of 3000 μS/cm across all sampling locations^63^. Based on total dissolved solids (TDS) measurements ranging from 199 to 370 mg/L, it has been noted that all samples are appropriate for irrigation. The predominant cations in descending order of abundance are Ca^2+^  > Mg^2^^+^ > Na^+^  > K^+^, with average concentrations of 53.8, 15.37, 16.06, and 2.6 mg/L, respectively. HCO_3_^−^ emerges as the most prevalent anion, followed by SO_4_^2−^, Cl^−^, CO_3_^2−^, and NO_3_^−^, with average concentrations of 189.5, 36.2, 23.36, 9.59, and 7.7 mg/L, respectively. These substantially elevated levels can be attributed to the dissolving of evaporite minerals and the cation exchange process with mineral clay^65–67^. Sodium concentrations range from 11 to 25.5 mg/L, all falling below the FAO standard irrigation limit, with higher levels observed in samples collected from the northern regions, indicating accumulation due to agricultural runoff. Potassium levels range from 0.1 to 4 mg/L, averaging at 2.6 mg/L, suggesting suitability for irrigation, with higher concentrations in the southern parts possibly attributable to weathering of potash feldspars or chemical fertilizers^64^.

**Table 4:** Descriptive statistics of pH and mean concentrations of surface water quality parameters in drinking and irrigation water sources in the Danube River basin, south of Hungary.

Sampling	S1	S2	S3	S4	S5	S6	S7	FAO	WHO^77^
Ca^2+^	54.45	54.15	54.41	53.93	53.59	53.72	53.11	400	75
Mg^2+^	16.13	15.59	15.7	15.31	15.25	14.76	14.5	60	50
Na^+^	16.56	16.68	16.56	15.63	15.87	15.57	15.27	919	200
Cl^−^	24.5	24.25	24.17	22.92	22.5	22.33	22.23	1036	250
SO_4_^2−^	37.25	37.08	37.08	36.67	35.08	35	34.85	960	250
HCO_3_^−^	195.42	188.33	193.33	190.83	190	188.33	181.15	610	120
CO_3_^2−^	9.17	10.08	8.08	10.42	8.75	9.17	10.23		350
T	14.53	14.06	14.93	14.48	15.51	15.44	16.21		
EC	467.92	466.67	462.08	464.58	463.75	464.17	451.15	3000	1500
TDS	272.5	263.38	251.88	255.71	296.36	254.7	257.44	2000	500
K^+^	2.58	2.69	2.6	2.5	2.74	2.64	2.57	2	12
NO_3_^−^	8.37	7.86	8.11	8.04	7.13	7.35	7.28		50
pH	8.14	8.16	8.16	8.14	8.16	8.18	8.13	8.5	8.5
TH	113.82	112.14	112.73	115.02	110.56	109.61	108.15		500
Fe^3+^	0.6	0.58	0.57	0.6	0.63	0.53	0.65	5	0.3
Mn^2+^	0.07	0.06	0.07	0.05	0.07	0.05	0.05	0.2	0.1

Calcium concentrations gradually increase from south to north, varying notably between 39 and 68 mg/L. Notably, all samplings exhibit calcium levels lower than the irrigation standard threshold, with these significant concentrations potentially stemming from the dissolution of gypsum and the dolomite’s incongruent dissolving, commonly known as dedolomitization^39,64,68^. The equation for the dedolomitization procedure is as follows:

14 $${\text{CaMg}}\left( {{\text{CO}}_{3} } \right)_{2} ({\text{s}}) + {\text{CaSO}}_{{4}} \cdot {\text{2H}}_{{2}} {\text{O}}({\text{s}}){\text{ + H}}^{ + } \rightleftharpoons {\text{CaCO}}_{3} ({\text{s}}) + {\text{Ca}}^{{{2} + }} {\text{ + Mg}}^{{{2} + }} + {\text{SO}}_{4}^{2 - } + {\text{HCO}}_{3}^{ - } + {\text{2H}}_{{2}} {\text{O}}$$

The concentrations of magnesium, ranging significantly from 12 to 24 mg/L, exhibited an increase from south to north within the study area, with all surface water samples falling below the FAO guidelines^63^. These deficient magnesium levels may be connected to the dissociation of ferromagnetic minerals and cation-exchange reactions^67,69^. Regarding bicarbonate concentrations, which varied from 125 to 255 mg/L, all samplings were confirmed to be under the acceptable level for irrigation, around 610 mg/L.

The chloride concentration of all samples varied between 15 and 40 mg/L, and all samples fell below the irrigation threshold. Notably, chloride levels were raised in the northern part of the research region owing to buildup caused by increasing human activities in agricultural and industrial/urban sectors.

Sulfate contents in surface water varied from 27 to 51 mg/L, with nearly all samples demonstrating sulfate contents below the FAO standard limit^63^. The presence of sulfates can be attributed to anthropogenic activities and/or the dissolution of gypsum (Eq. 15) and/or anhydrite minerals (Eq. 16), as outlined by the following equations:

15 $${\text{CaSO}}_{{4}} \cdot {\text{2H}}_{{2}} {\text{O}} \rightleftharpoons {\text{Ca}}^{{{2} + }} {\text{ + SO}}_{4}^{2 - }$$

16 $${\text{CaSO}}_{{4}} \rightleftharpoons {\text{Ca}}^{{{2} + }} + {\text{SO}}_{4}^{2 - }$$

Figure 1S displays the distribution maps illustrating the physicochemical parameters along with the heavy metals across the study area.

### Surface water facies and source determination

#### Surface water categories

**Figure 3:**
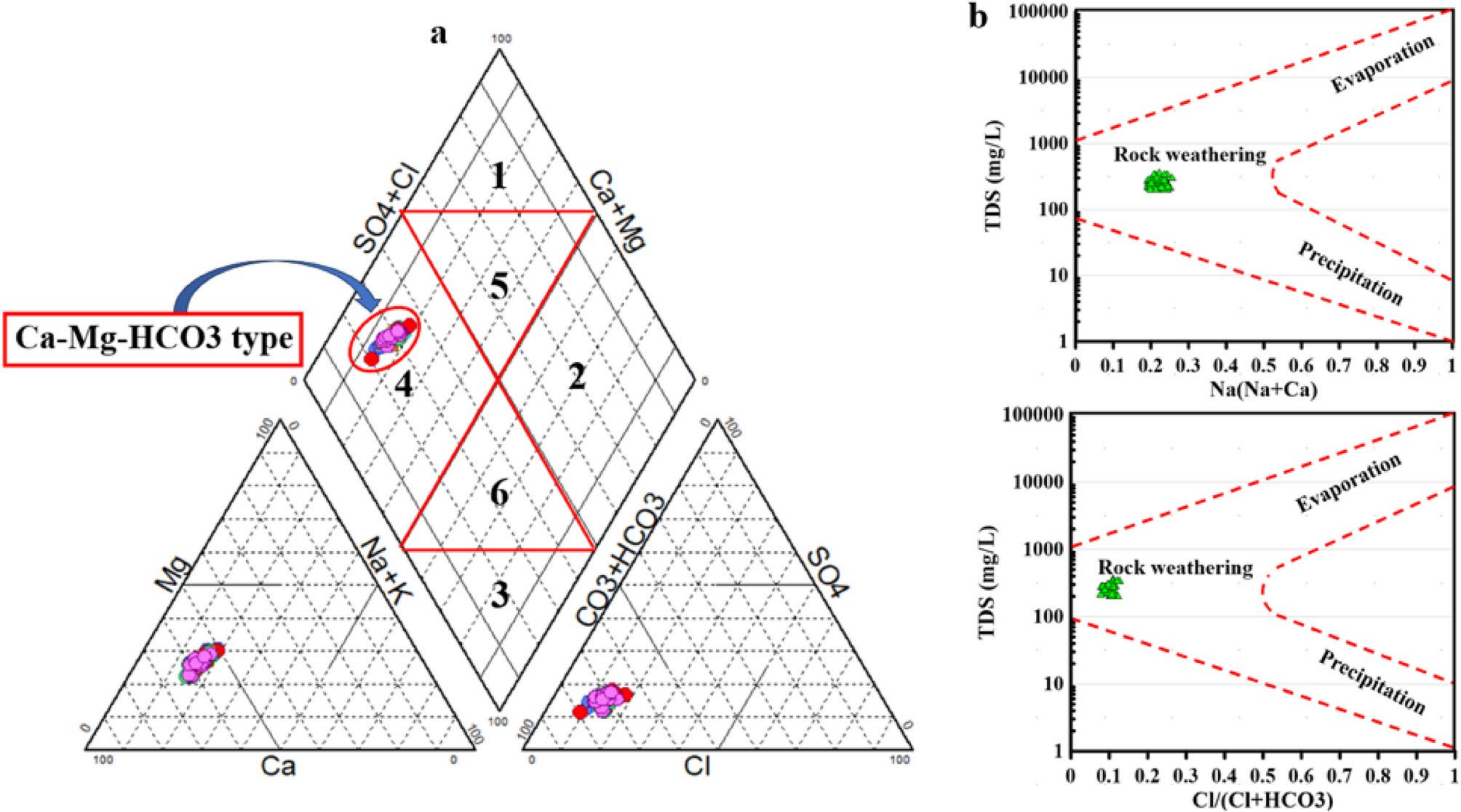
Surface water facies according to Piper diagram (**a**) and geochemical controlling mechanisms according to Gibbs diagram (**b**).

Surface water in Hungary’s lower Danube River basin could be categorized into distinct water facies by classifying ions based on their dominance levels. Several diagrams, such as Piper’s diagram, may assist in identifying chemical water facies^70^. The Piper diagram enabled Danube River surface water to be classified into a single chemical water type, precisely a Ca–Mg–HCO_3_^−^ type (Fig. 3a). These water facies are primarily characterized by the predominant proportions of bicarbonate, indicating continuous recharge, whereas cations show a general tendency towards the calcium pole in most samples.

On the other hand, the Gibbs diagram, widely utilized to analyze the connection between the composition of water and lithological features, indicates all SW samples belong to the area of rock weathering dominance due to water–rock interactions along the flow path from south to north (Fig. 3b). This shows that solutE−enriched water resulting from carbonate mineral dissolution and/or ion exchange with silicate minerals, as well as irrigation water return, have the most significant impact on surface water composition^65,71,72^. Nevertheless, these samples also show a preference for the rock weathering pole, indicating the influence of cation-exchange mechanisms.

#### Ion exchange

**Figure 4:**
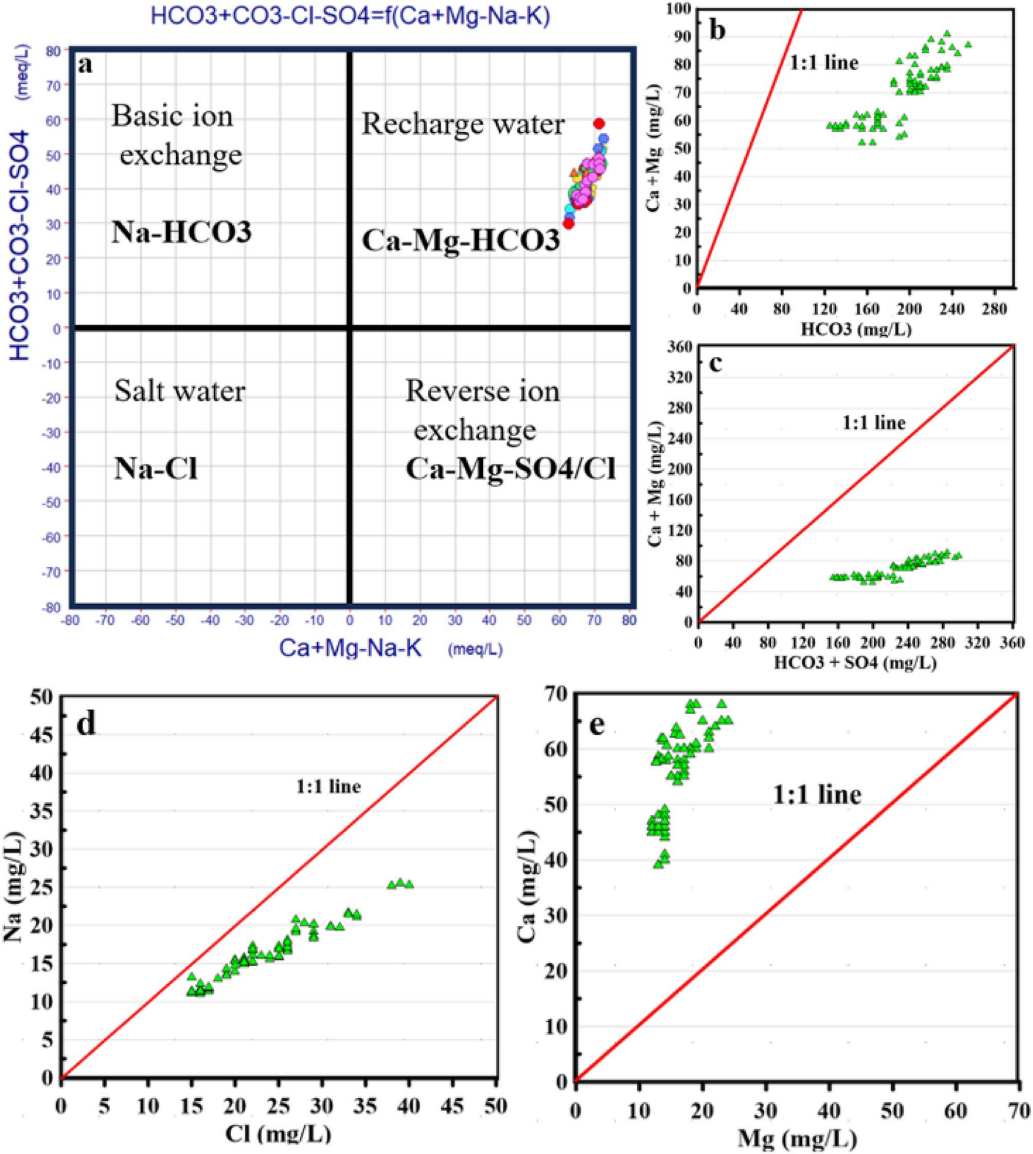
Relationships between the main cations and anions in the sample water using stoichiometry: (**a**) Ca + Mg–(Na + K) versus (HCO_3_-(SO_4_ + Cl), (**b**) Ca^2+^ + Mg^2+^ versus HCO_3_, (**c**) Ca^2+^ + Mg^2+^ versus HCO_3_^−^ + SO_4_^2−^, (**d**) Na^+^ versus Cl^−^, (**e**) Ca^2+^ versus Mg^2+^.

Statistical analysis, focusing on the correlations and proportions of various main ions, was applied to determine the key mechanisms driving surface water chemistry in the research region (Fig. 4). In aquifer systems where clay minerals are relatively abundant, ion exchange is pivotal in controlling surface water mineralization. Clay minerals often balance their electrical charge by storing monovalent cations (such as Na^+^ and K^+^) while releasing bivalent cations (such as Ca^2+^ and Mg^2+^), or vice versa.

The graph of [(Ca_2_^+^ + Mg^2+^)−(Na^+^ + K^+^)] versus [HCO_3_^–^−(SO_4_^2−^ + Cl^−^)] demonstrates that all surface water samples fall within the Ca–Mg–HCO_3_^−^ class, indicating that the river is continuously recharged and that Ca, Mg, and HCO_3_^−^ ions dominate the water chemistry throughout the flow channel (Fig. 4a). This underscores the importance of ion exchange in surface water mineralization in the Danube River^72^, a phenomenon closely tied to cationic interactions with minerals of clay observed in the examined river network^73^. Throughout this procedure, Ca^2+^ and Mg^2+^ ions, which had previously been adsorbed onto the outermost layer of clay minerals, interchange with the Na^+^ and K^+^ ions in surface water. This ionic exchanging mechanism can be represented by the following Eq. (17) ^72,74^.

17 $$\left( {{\text{Ca}}^{{{2} + }} ,{\text{ Mg}}^{{{2} + }} } \right) - {\text{Clay }} + { 2}\left( {{\text{Na}}^{ + } ,{\text{ K}}^{ + } } \right) \rightleftharpoons \left( {{\text{Na}}^{ + } ,{\text{ K}}^{ + } } \right) - {\text{Clay }} + \, \left( {{\text{Ca}}^{{{2} + }} ,{\text{ Mg}}^{{{2} + }} } \right)$$

Silicate weathering or ion exchange processes are responsible for reducing the (Ca^2+^  + Mg^2+^)/HCO_3_^−^ ratio and the Ca^2+^ + Mg^2+^ / (HCO_3_^−^ + SO_4_^2−^) ratio to values below 1 (Fig. 4b,c). In ion exchange, Ca^2+^ + Mg^2+^ ions are removed from the water while Na^+^ and K^+^ ions are added^64^. Plotting the summation of Ca^2+^ + Mg^2+^ ions against HCO_3_^−^ + SO_4_^2−^ ions on a linear graph helps identify the source of Ca^2+^ + Mg^2+^ in water samples^39,64^. Ratios below 0.5 may indicate ion exchange or bicarbonate enrichment as the leading causes of calcium and magnesium depletion. All samples fall below the 1:1 line, indicating higher proportions of HCO_3_^−^ than Ca^2+^ + Mg^2+^, suggesting sources of HCO_3_^−^ ions other than the dissolution of calcite and dolomite.

The linear relationship between Na^+^ and Cl^−^ (Fig. 4d) reveals an unbalanced presence of these ions in water samples. This correlation suggests that halite dissolution does not significantly contribute to sodium and chloride ions, and the dominance of chloride ions over sodium ions reflects anthropogenic sources of Cl^−^ ions through agricultural drainage and industrial activities. Most samples shift to the right of the 1:1 line, indicating additional Cl^−^ sources or attributing to Na^+^ removal through recharge water (Fig. 4d).

Examining the ionic relationship between Ca^2+^ + Mg^2+^ (Fig. 4e) shows that all samples fall above the 1:1 line, indicating an excess of calcium ions and a depletion of magnesium ions. This implies that the river in the research area receives Ca ions from different sources rather than dolomite dissolution, which could be from calcite dissolution or agricultural drainage rich in Ca ions from calcium fertilizers.

#### Chloro-alkaline indices

**Figure 5:**
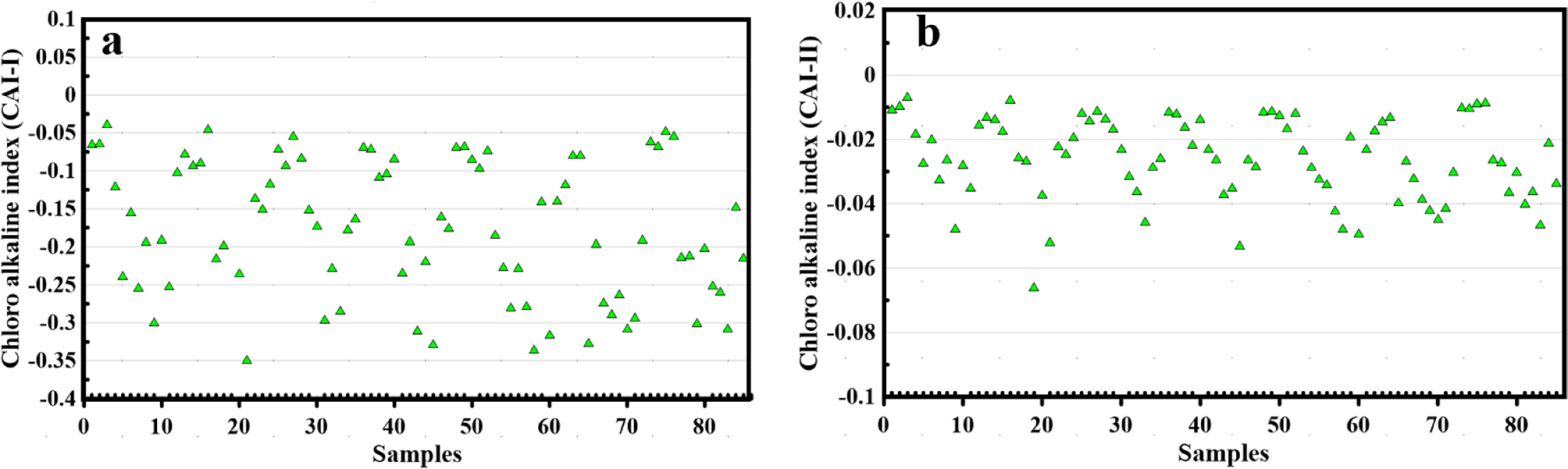
Relationships between Samples versus CAI-I, and (**a**) Samples versus CAI-II (**b**).

The chloro-alkaline method was employed to discern the predominant mechanism, whether ion exchange or reverse ion exchange, influencing the minerals within the lower Danube River Basin. Overall, the Chloro-alkaline indices (CAI) values, including CAI-I and CAI-II, exhibited negative values across all water samples (Fig. 5a,b). This negativity signifies a notable inclination towards ion exchange, particularly between K^+^ and Na^+^ in the research area’s surface water, and between Ca^2+^ and Mg^2+^ within the neighboring geological formations. Through analysis of various ionic ratio relationships, it was determined that ion exchange predominantly governed the surface water chemistry in the area.

#### Geochemical modeling and mineral saturation state

**Figure 6:**
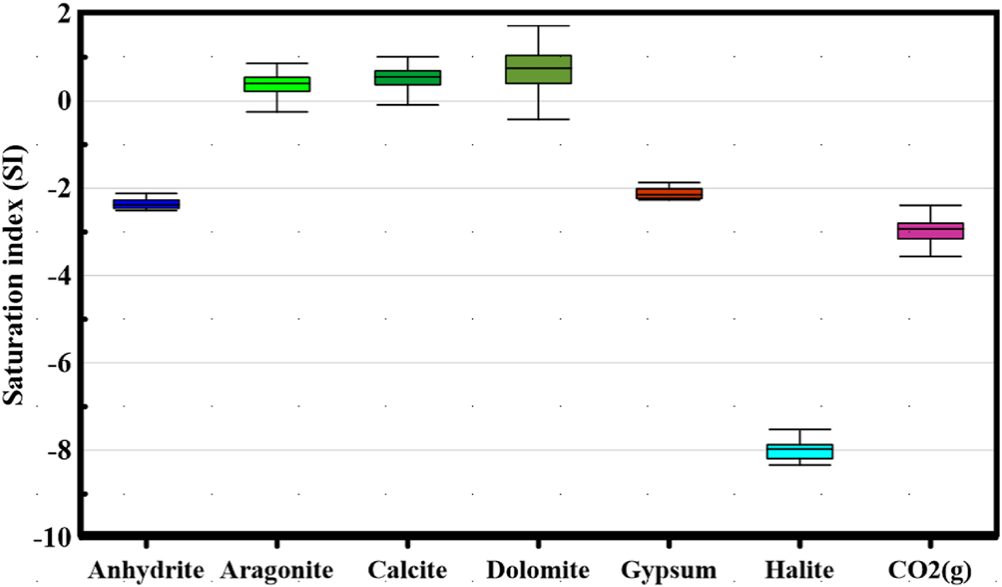
Mineral saturation state showing the ability of precipitation and dissolution of minerals in Danube River.

We used the PHREEQC model^75^, to analyze mineral levels, saturation metrics, and surface water’s (SW) ability to dissolve or precipitate minerals. The model generated saturation metrics for significant minerals such as dolomite, calcite, gypsum, halite, aragonite, and anhydrite, along with the potential pressure of CO_2_. The inputs included pH, T (°C), (TDS), (EC), and main anions and cations (Fig. 6). The study revealed a negative partial pressure of CO_2_ below saturation levels within the lower Danube River basin, suggesting a deficit in water recharge relative to extraction from the nearby aquifer. Additionally, it was observed that carbon dioxide concentrations diminish in tandem with the directional flow of water, attributable to a concurrent reduction in surface water recharge in the same direction.

### Cluster and correlation matrix analysis

**Figure 7:**
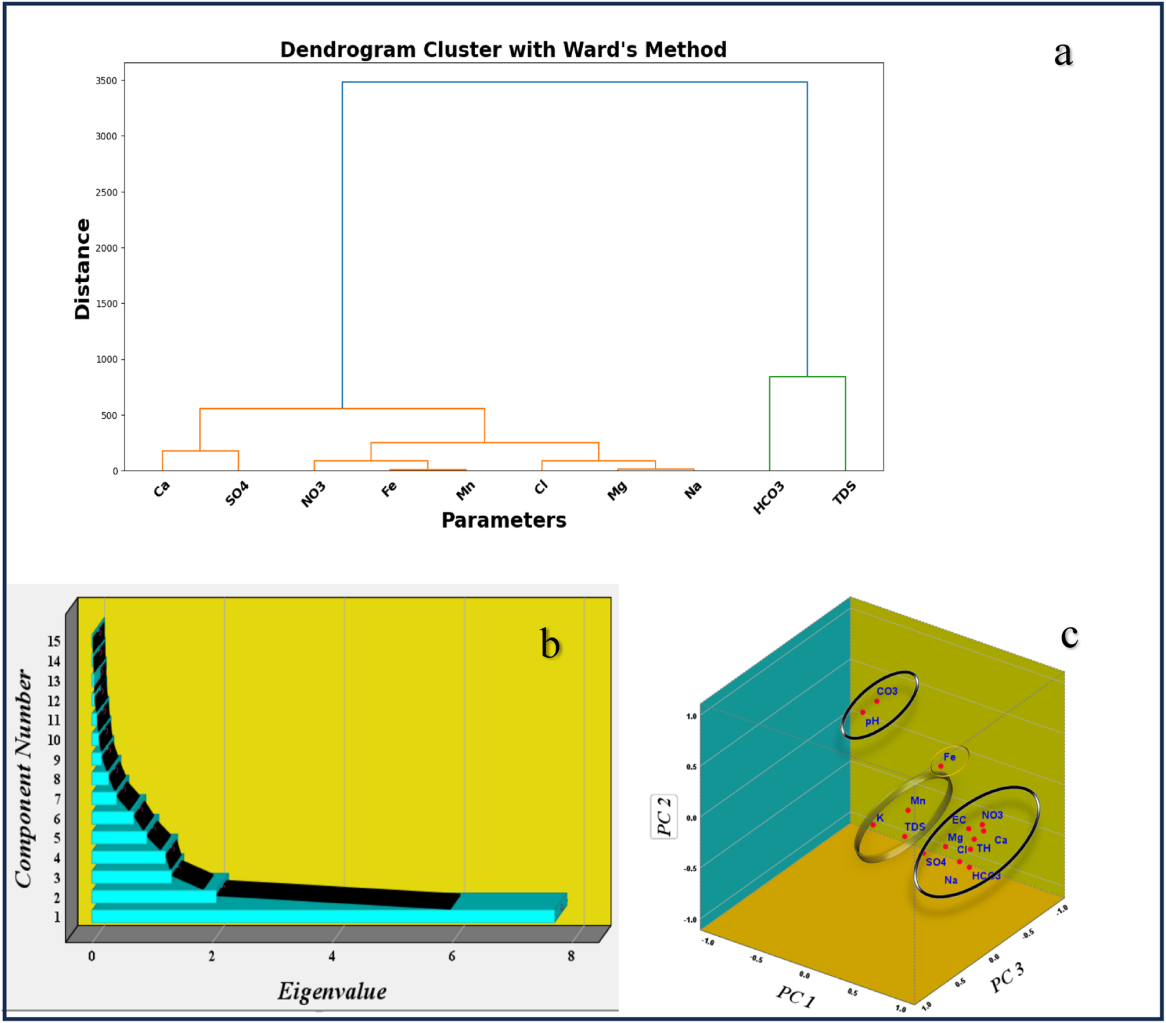
Multivariate statistical analysis: (**a**) Cluster dendrogram for variables showing the number of clusters; (**b**) Scree plot showing optimum number of component and (**c**) PCA scores for PC2 versus PC1 versus PC3.

We used the Euclidean distance along with Ward linkage technique to determine the similarity of SW samplings in the research region. Figure 7a shows a dendrogram that categorizes the numerous physicochemical parameters detected in the gathered surface water samples. Z-scores were computed for every factor and used in statistical analysis. All factors were logarithmically transformed, and the data distribution was almost identical to normal. Within the dendrogram depicting eight physicochemical parameters (Ca^2+^, SO_4_^2−^, NO_3_^−^, Cl^−^, Mg^2+^, Na^+^, HCO_3_^−^, TDS) and two heavy metals (iron and manganese), three primary clusters were identified. Each height parameter was then separated into two groups based on TDS and HCO_3_^−^ levels. Cluster1 encompassed Ca^2+^ and SO_4_^2−^. Conversely, cluster 2 comprised NO_3_^−^, Fe^3+^, Mn^2+^, Mg^2+^, Na^+^ and Cl^−^ representing predominantly carbonate components. Finally, cluster 3 illustrated varied associations of all metrics with salinity and HCO_3_^−^ in the region, indicating diverse sources influencing surface water composition.

The Pearson correlation matrix (Table 5) reveals complex interactions among water quality parameters^21^. At *p* < 0.01, calcium strongly & positively correlated with magnesium, sodium, chloride, sulfate, bicarbonate, and nitrate, suggesting common sources or geochemical behaviors. Magnesium strongly correlated with Na^+^, Cl^−^, SO_4_^2−^, HCO_3_^−^, and NO_3_^−^, indicating similar sources such as mineral dissolution or anthropogenic inputs. Sodium strongly correlated with Cl^−^ (r = 0.970), SO_4_^2−^ (r = 0.860), HCO_3_^−^ (r = 0.803), and NO_3_^−^ (r = 0.695), reflecting geochemical processes and anthropogenic activities like agricultural runoff and wastewater discharge. Chloride’s strong correlations with Na^+^, Mg^2+^, and Ca^2+^ suggest associations from NaCl, MgCl_2_, and CaCl_2_ sources. Sulfate’s strong correlations with Mg^2+^, Na^+^, Cl^−^, Ca^2+^ and HCO_3_^−^, point to sources like mineral dissolution (e.g., gypsum) and industrial pollution. Water alkalinity (HCO_3_^−^) is higher at all locations, perhaps due to CO_2_ dissolution from the atmosphere and carbonate mineral weathering^21,22,58^. Total Dissolved Solids (TDS) moderately correlate with Mg^2+^, Na^+^, Cl^−^, SO_4_^2−^, and HCO_3_^−^, reflecting these ions’ contributions to overall dissolved substances. Iron (Fe) negatively correlated with Mg and SO_4_, indicating different sources or processes affecting iron levels. Manganese (Mn) has very weak correlations with all other ions, suggesting it behaves independently in the water system due to distinct sources or geochemical behavior.

**Table 5:** Pearson correlation matrix showing the association between measured attributes.

	Ca^2+^	Mg^2+^	Na^+^	Cl^−^	SO_4_^2−^	HCO_3_^−^	TDS	NO_3_^−^	Fe	Mn
Ca^2+^	1	0.757**	0.844**	0.876**	0.628**	0.854**	0.244*	0.827**	0.065	0.092
Mg^2+^		1	0.920**	0.877**	0.930**	0.681**	0.305**	0.599**	− 0.286**	0.063
Na^+^			1	0.970**	0.860**	0.803**	0.340**	0.695**	− 0.125	0.060
Cl^−^				1	0.801**	0.819**	0.330**	0.766**	− 0.014	0.024
SO_4_^2−^					1	0.596**	0.259*	0.463**	− 0.460**	− 0.022
HCO_3_^−^						1	0.280**	0.771**	0.029	0.118
TDS							1	0.209	− 0.073	0.001
NO_3_^−^								1	0.163	0.164
Fe									1	0.126
Mn										1

### Principal component analysis (PCA)

In this investigation, PCA was performed to pinpoint the factors that impact water quality indicators in the Danube River. First, these factors were identified based on their eigenvalues, keeping those components with eigenvalues above 1 using the Kaiser criterion. This approach ensures that only factors explaining a significant variance are considered.

Varimax rotation was employed to help the components be understood. This method aims to maximize the variance of factor loadings squared across variables, simplifying the components’ structure and helping to identify which variables are strongly linked to each element. It was chosen to distinguish between variables and provide insight into the processes influencing water quality.

In this investigation, we utilized the PCA to ascertain the likely sources of the investigated water quality indicators. Through PCA, we identified four key factors that exert significant control, each with eigenvalues exceeding one based on scree plot (Fig. 7b), across the datasets collected during the sampling period. Detailed findings from the PCA analysis are presented in Table 6. Our investigation applied PCA with Kaiser normalization (≥ 0.75) to extract four principal components across all sampling points. These components were recognized as influential factors shaping the condition of Danube surface water in our investigation area. The dataset yielded by this analysis was characterized by four principal components, collectively explaining 82.098% of the overall variance with eigenvalues greater than 1 and the PC1, PC2, and PC3 were vizualized on 3D plot (Fig. 7c). Principal Component 1 (PC1) accounted for 49.76% of the total variance observed, with an eigenvalue of 7.47. Variables associated with PC1 emerged as crucial indicators, reflecting both anthropogenic activities and natural phenomena in shaping surface water chemistry. Notably, variables such as Ca^2+^, Mg^2+^, Na^+^, Cl^−^, SO_4_^2−^, HCO_3_^−^, EC, NO_3_^−^, and TH demonstrated strong positive loadings.

**Table 6:** Varimax rotated factor of principal component analysis.

Measured parameters/PCA analalysis	PC1	PC2	PC3	PC4
Rotated component matrix				
Ca^2+^	0.945	− 0.053	− 0.149	− 0.003
Mg^2+^	0.897	0.011	0.347	0.040
Na^+^	0.933	− 0.199	0.190	0.003
Cl^−^	0.957	− 0.130	0.062	− 0.013
SO_4_^2−^	0.791	− 0.002	0.526	− 0.031
HCO_3_^−^	0.843	− 0.414	− 0.084	− 0.049
CO_3_^2−^	− 0.076	0.940	− 0.026	− 0.044
EC	0.926	0.054	0.043	− 0.086
TDS	0.308	− 0.197	0.110	− 0.598
K^+^	0.127	− 0.042	0.321	0.774
NO_3_^−^	0.825	− 0.104	− 0.302	0.069
pH	− 0.102	0.904	0.145	− 0.009
TH	0.985	− 0.029	0.047	0.014
Fe^3+^	− 0.028	− 0.096	− 0.904	0.033
Mn^2+^	0.093	− 0.179	− 0.246	0.518
Eigenvalues	7.464	2.029	1.577	1.245
% of variance	49.759	13.527	10.511	8.300
Cumulative %	49.759	63.287	73.798	82.098

Conversely, PC2 accounted for 13.53% with an eigenvalue of 2.029, representing the influence of human activities on surface water resources, exhibiting a positive correlation with pH and carbonate (CO_3_^2−^) concentrations. This finding suggests that localized and limited anthropogenic activities within the study area can affect surface water quality by altering pH and carbonate levels^76^. While silicate weathering is still the dominant governing mechanism for pH and carbonate levels, it is essential to recognize that human activities such as farming procedures and industrial emissions may bring new ions into surface water. These newly added ions may interact with existing geochemical processes, possibly generating changes in pH and carbonate levels. Consequently, natural and human activities both have an impact on surface water quality in the studied area. PC3 was shown to explain 10.51% of the total observed variance, with an eigenvalue of 1.58. Notably, it showed a negative connection only with iron. Conversely, Component PC4 accounted for 8.300% of the total variance, characterized by an eigenvalue of 1.24. This component exhibited a positive correlation with Potassium (K^+^) and Manganese (Mn^2+^), while displaying a negative correlation with Total Dissolved Solids (TDS).

### Drinking water quality index (DWQI)

**Figure 8:**
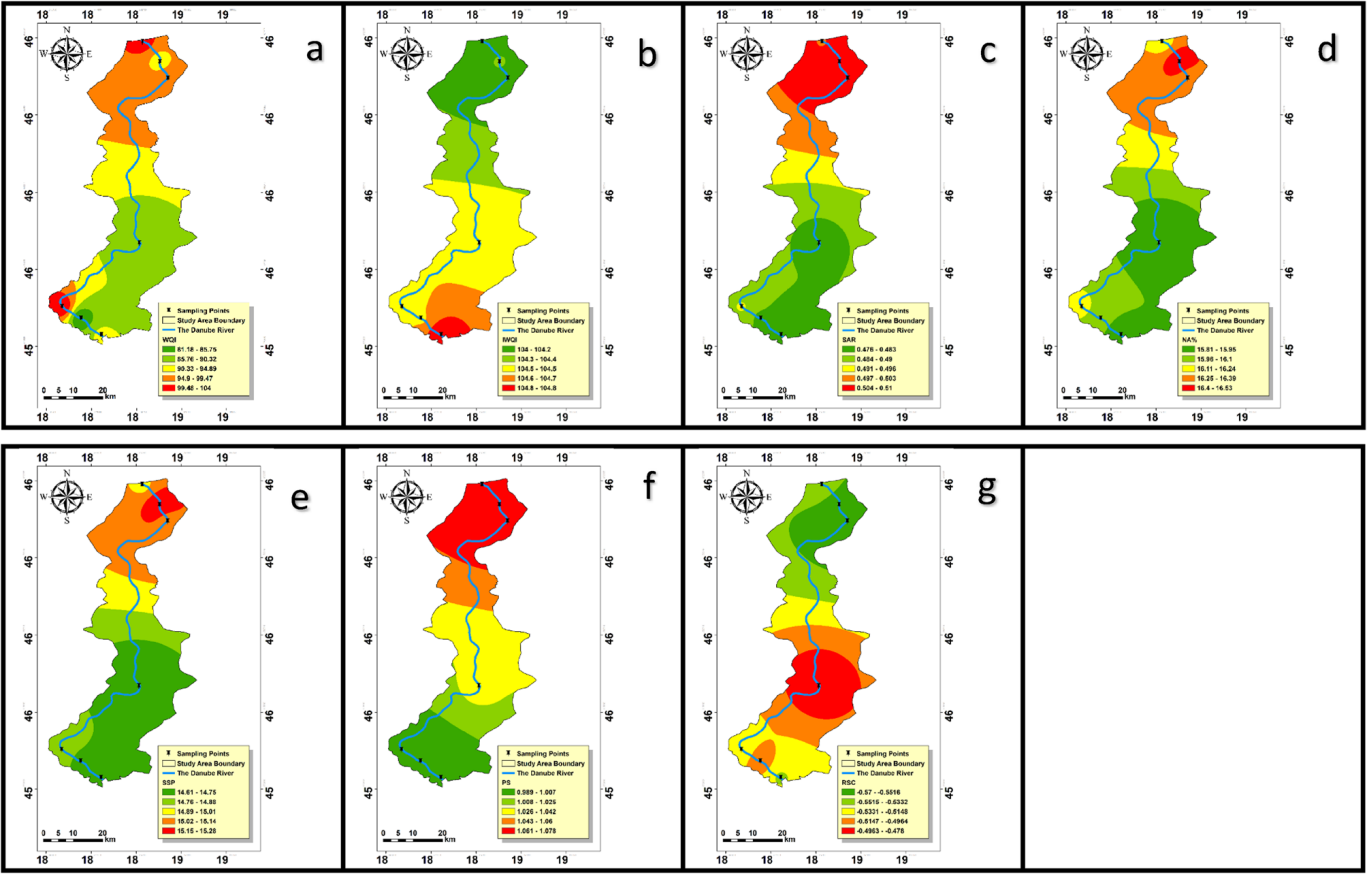
The spatial variation maps of the WQI and IWQIs for the lower Danube River basin: (**a**) WQI for drinking purposes (**b**) IWQI for irrigation purposes, (**c**) SAR, (**d**) Na %, (**e**) SSP, (**f**) PS, and (**g**) RSC.

The aquatic environment standards established by WHO in 2017^77^ were applied in computing the DWQI scores about drinking water quality. The evaluation of the DWQI throughout the seven sampling sites within the Lower Danube River Basin showed alarming findings, revealing variations in water suitability for different uses. Specifically, sampling locations S2, S3, S4, S6, and S7 exhibited WQI values falling within the range of 76–100, indicating water quality suitable for drinking purposes. However, contrasting results were observed in sampling locations S1 and S5, where WQI values exceeded 100, designating restricted use for drinking due to poorer water quality. This nuanced analysis sheds light on the diverse water quality characteristics within the basin and underscores the importance of tailored management approaches for different areas. Factors contributing to the inappropriateness of the water for consumption include elevated levels of Iron and bicarbonate contaminants exceeding the permissible limits of the WHO 2017^77^. These results draw attention to the crucial significance of regular surveillance, strict regulatory measures, and stakeholder collaboration to protect water resources, reduce pollution sources, and ensure safe drinking water for the Lower Danube River Basin population. Resolving those issues requires a multidimensional strategy that includes severe pollution reduction policies, effective wastewater treatment techniques, and comprehensive watershed management plans to protect the basin’s ecological integrity and public health (Fig. 8a).

#### Comparison between water quality in Danube River with different rivers

In our research, DWQI was performed or computed based on WHO in 2017. When assessing water quality at seven sampling sites in the Lower Danube River Basin, we discovered varying suitability levels for drinking purposes. Specifically, sites S2, S3, S4, S6, and S7 had WQI values ranging from 76 to 100, indicating that the water quality was suitable for drinking. In contrast, sites S1 and S5 showed WQI values above 100, suggesting that drinking water is unsuitable due to poorer water quality. The main factors contributing to this unsuitability were levels of iron that exceeded WHO standards.

Comparing our findings with similar global studies, such as the one by Ghimire^78^ on rivers in Damak, Jhapa, Nepal, highlights common water quality index across different regions. In both studies, high iron concentrations were a significant factor in water quality assessments. For instance, Ghimire et al. found that the water quality in several rivers was unsuitable for drinking and other uses due to iron levels exceeding permissible limits. This comparison highlights the significance of iron, as a contaminant impacting water quality and underscores the importance of robust monitoring and management strategies.

### Indices of water quality of irrigation (IWQIs)

The evaluation of the soil’s water quality and its impact on the crops specifications demands an investigation of numerous key parameters. These indications might include specific chemical markers^79^ or a group of indicators^80,81^. Decision-makers may develop effective irrigation water management strategies by understanding the results of these indicators. This is exactly the topic of the current research, which investigates the following indicators. Water quality was classified for irrigation purposes using known parameter values, and all six IWQIs listed in Table 2S.

### IWQI and categorization

The IWQI, computed applying the formula given in Table 2S ^39,81^, is a critical tool for assessing water quality in the research area, especially for agricultural irrigation. This index meticulously measures several water composition characteristics, which may considerably impact irrigation appropriateness and soil condition. The full evaluation divides water quality into five separate groups^63^, each with specific implications for irrigation practices and soil condition. Interestingly, the distribution of these classes across the dataset reveals that 100% of samples indicate no restrictions for irrigation. This meticulously delineated distribution is visually (Fig. 8b), emphasizing the consistency of water quality throughout the research region.

The numerical results attributed to the IWQI vary widely, ranging from 99.6 to 107.6, with an average of 104.36. This diversity highlights the many elements that influence water quality. Geospatial analysis (Fig. 8b), identifies specific areas with compromised water quality, raising concerns about their suitability for irrigation and potential impacts on soil health. Notably, all samples exhibit water quality levels classified as excellent and suitable for irrigation with no restrictions. This finding holds significant implications, as such conditions may adversely affect soil’s permeability, availability of nutrients, and total agricultural production. Therefore, based on the IWQI findings, there is no necessity for and effective cost associated with water purification and fertilization before irrigation in the investigated locations along the Danube River.

#### Impact on soil composition

Agricultural water quality has a considerable impact on the composition of soil, particularly impacting permeability, filtration rate, and ventilation owing to its specific chemical qualities^82^. Despite these characteristics, sodium ion concentration is particularly critical. Excessive sodium’s levels affect soil filtration systems by displacing calcium and magnesium ions via adsorption^64^. In assessing water suitability for irrigation, indicators such as the Sodium Percentage (Na%), the Sodium Adsorption Ratio (SAR), and Soluble Sodium Percentage (SSP) serve as valuable tools. These indices, calculated based on sodium, magnesium, and calcium levels, not only help identify areas susceptible to soil deterioration, but also provide a basis for informed soil management techniques.

The range of values for SAR, Na%, and SSP spans a diverse spectrum. Minimum values recorded are 0.37, 13.75, and 12.52, respectively, while upper limits peak at 0.68, 18.72, and 17.59. Remarkably, average values for these indices stand at 0.49, 16.11, and 14.88, respectively. Classification based on these indices reveals that 100% of samplings are classified as good for irrigation (Fig. 8c,d,e). This detailed analysis highlights the intricate interchange between soil integrity, agricultural yield, and irrigation water quality. The correlation between levels of sodium and soil condition emphasizes that expensive water treatment techniques may not be necessary in specific regions where current water quality poses no negative impacts on soil structure and fertility.

#### Potential salinity index (PSI)

The Potential Salinity Index (PSI), which is generated from chloride and sulfate ion concentrations, is applied to assess the appropriateness of irrigation water for farming fields ^64^. The PS index divides findings into three levels: excellent to good (PS < 3.0), good to injurious (PS = 3.0–5.0), and injurious to unsatisfactory (PS > 5.0). The PS index findings show a notable trend: all water samples in the collection, accounting for 100% of the overall, were classed as acceptable to excellent for irrigation (Fig. 8f). This discovery reaches beyond only assessing water quality; it also shows possible benefits for the soil’s structure, availability of nutrients, and agricultural output.

The ramifications of such a categorization are significant, suggesting that intervention methods to maintain soil sustainability may be ineffective. The accumulated consequences of using samplings with injurious to unsatisfactory PS scores might jeopardize soil conditions, diminish crop yields, and impede agricultural sustainability. However, in the present investigation, this situation does not occur.

#### Precipitation of alkali elements and RSC

Excess carbonates and bicarbonates may negatively impact irrigation water quality when combined with Ca and Mg. This imbalance may cause precipitation of alkali metals, especially Ca and Mg, degrading the irrigation water’s quality. Carbonate minerals precipitate Ca and Mg ions, leading to increased sodium ion concentrations and Sodium Adsorption Ratio (SAR)^83^. In arid environments, high Residual Sodium Carbonate (RSC) levels can disrupt soil physical properties. This process often results in the dissociation of organic matter, leaving a noticeable black mark on the soil’s surface after desiccation^84,85^.

This work used RSC calculations to predict Ca^2+^ and Mg^2+^ precipitation on soil particle surfaces is widely recognized and applied in arid and semi-arid regions, where greater levels are related with soil salinization^86^. Our investigation, conducted in a wet climate, revealed a notable classification based on RSC values, as depicted in Fig. 8g. In accordance to this categorization structure, irrigation water with an RSC more than 2.5 is inappropriate for irrigation, but water with an RSC less than 1.25 is considered acceptable. Water has an RSC of 1.25–2.5, making it dubious for irrigation usage^83^. A remarkable finding of our study is that all samplings had an RSC value of less than 1.25. This cumulative observation significantly supports the usefulness and safety of surface water for irrigation.

### Human health risk assessment

#### Non-carcinogenic health risk

**Figure 9:**
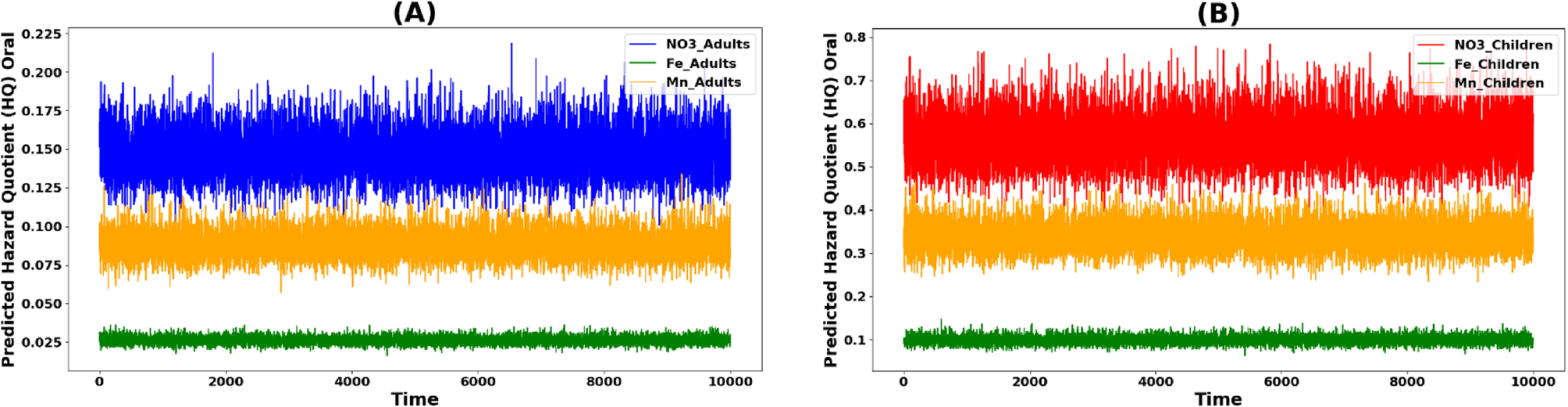
Predicted oral hazard quotient: (**A**) Adults, (**B**) Children.

The daily intake of Iron (Fe^3+^), Manganese (Mn^2+^), and Nitrate (NO_3_^−^) through oral ingestion relies on key parameters sourced from USEPA (2004)^57,61^. Figure 9 illustrates the non-carcinogenic risk assessment of these elements in surface water for both adults and children across various sampling sites, while Table 3S presents the hazard quotient (HQ) values and resultant non-carcinogenic risks. In children, HQ values for Fe^3+^, Mn^2+^, and NO_3_^−^ range from 0.002 to 0.477, 0.096 to 1.678, and 0.190 to 0.927, respectively, with means of 0.092, 0.281, and 0.523. For adults, corresponding HQ values span from 0.001 to 0.125, 0.025 to 0.439, and 0.050 to 0.243, with means of 0.024, 0.074, and 0.173, respectively (Table 4S). Surface water contaminants pose a significantly higher mean hazard index (HI) for children compared to adults, indicating a substantial non-carcinogenic risk within the study area (Table 4S)). Notably, NO_3_^−^ and Mn^2+^ concentrations play a pivotal role in contributing to non-carcinogenic risk, followed by Fe^3+^. Moreover, HI values for children via oral exposure pathway exceed those of adults for the studied elements, and the total hazard index for non-carcinogenic risk is 2.1 times higher for children than for adults (Table 4S). Monte Carlo was utilized to forecast the HQ (oral) values of Fe^3+^, Mn^2+^, and NO_3_^−^ for both adults and children, employing 10,000 iterations in Python. The simulated outcomes of the Monte Carlo indicated that the projected HQ values for Fe^3+^, Mn^2+^, and NO_3_^−^ remained below the standard limits (HQ < 1) for adult and children (Fig. A, B). Consequently, according to the Monte Carlo simulation, the estimated exposure levels are improbable to pose a significant health risk for either population via oral pathways. Nonetheless, it is crucial to acknowledge that risk assessments typically rely on conservative assumptions and uncertainties in the available data. Thus, it is imperative to persist in monitoring exposure levels and updating risk assessments as new information emerges (Fig. 9).

## Conclusion

The assessment of water quality is critical in ensuring effective water resource utilization, human consumption, and environmental stability. In Hungary’s lower Danube River basin, a comprehensive range of approaches were used to analyze, replicate, and predict the quality of surface water for irrigation. These approaches incorporated the use of water quality indicators (WQIs), complex statistical analyses with many variables, and the integration of geographic information systems (GIS). Detailed physicochemical investigations were conducted on surface water samples to uncover their compositional attributes. Results indicated a sequence of ion abundance with Na^+^  > Ca^2+^  > Mg^2+^  > K^+^ and SO_4_^2−^ > HCO_3_^−^  > Cl^−^. Physicochemical analyses of surface water samples revealed primarily Ca-Mg-HCO_3_ is the dominant water types. Principal component analysis (PCA), ionic ratios and piper, chloro alkaline index, Chadha, and Gibbs diagrams identified three distinct water characteristics influenced by processes such as water–rock interaction, dolomite dissolution, evaporation, ion exchange, and human activities. The and geochemical modeling showed strong ability of Danube River water to dissolve gypsum, halite, and anhydrite (SI < 0). and precipitate aragonite, dolomite and calcite with saturation index (SI) value greater than 0 along its flow path. The irrigation water quality Index (IWQI = 99.6–107.6), sodium adsorption ratio (SAR = 0.37–0.68), sodium percentage (Na% = 13.7–18.7), soluble sodium percentage (SSP = 12.5–17.5), Potential Salinity (PS = 0.73–1.6), and Residual Sodium Carbonate (RSC = − 1.27–0.58) values were used, indicating mostly acceptable quality with some limitations. Danube River water was not suitable for drinking purposes based on WQI value (WQI = 81–104). Oral exposure of children to certain components showed a higher Hazard Index (HI > 1) compared to adults, indicating a 2.1 times higher overall non-carcinogenic risk hazard index. However, Monte Carlo simulation demonstrated negligible health hazards from iron, manganese, and nitrates for both age groups. These findings are valuable for water quality management decisions, contributing to long-term resource The application of modern approaches, such as WQIs, detailed statistical analysis, and GIS integration, provides a complete view of surface−water quality changes. Moreover, the discovery of various water categorizations, the explication of fundamental processes, and the complete assessment of irrigation-related indicators all contribute significantly to water quality control in comparable geographical settings. This research lays the groundwork for potential water resource control and provides prospective measures to promote both human and environmental well-being.

### Supplementary Information

Supplementary Information.

## Data Availability

Most of the data is available in tables and figures inside the manuscript and supplementary. The concentration of ions and metals in each sample is available upon request and the corresponding authors are responsible to provide these data.
