# Supplementary material for: Assessing surface water quality in Hungary’s Danube basin using geochemical modeling, multivariate analysis, irrigation indices, and Monte Carlo simulation
Source: Sci Rep. 2024 Aug 11;14:18639. doi: 10.1038/s41598-024-69312-8 (PMC11317494; doi:10.1038/s41598-024-69312-8)
Supplement: Supplementary file 1 — Supplementary Information. [file 41598_2024_69312_MOESM1_ESM.docx]

**Assessing Surface Water Quality in Hungary’s Danube Basin using Geochemical Modeling, Multivariate Analysis, Irrigation Indices, and Monte Carlo Simulation**

| 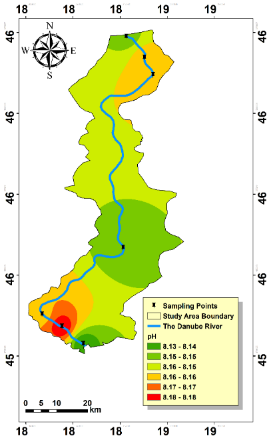  a text here | 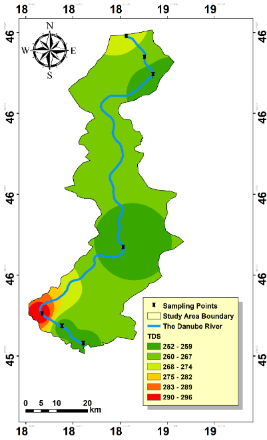  b text here | 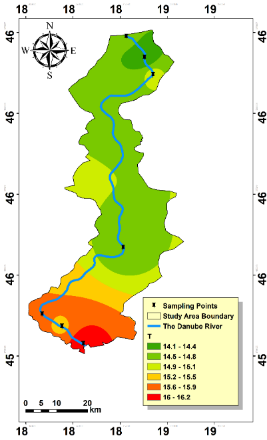  c text here | 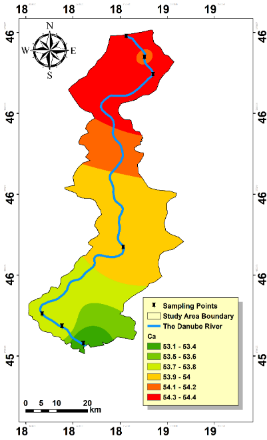  d text here |
| --- | --- | --- | --- |
| 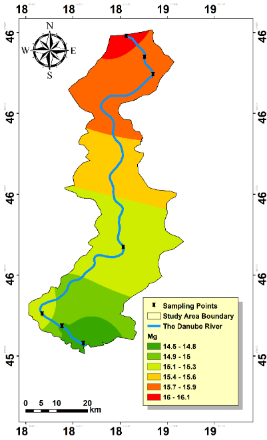  e text here | 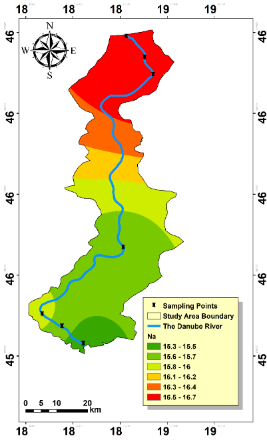  f text here | 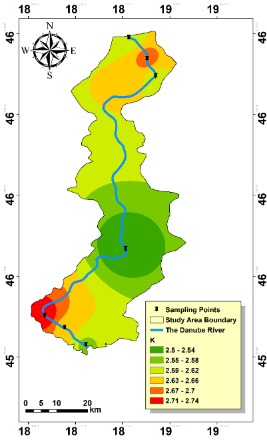  g text here | 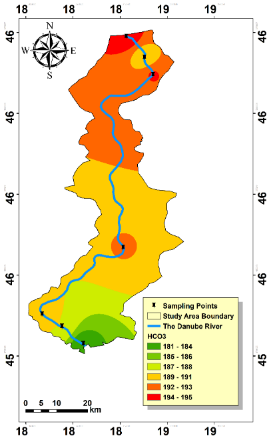  l text here  h text here |
| 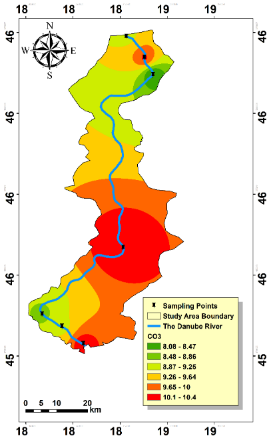  i text here | 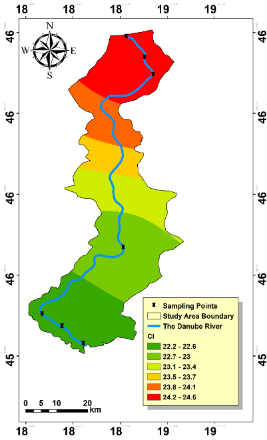  j here | 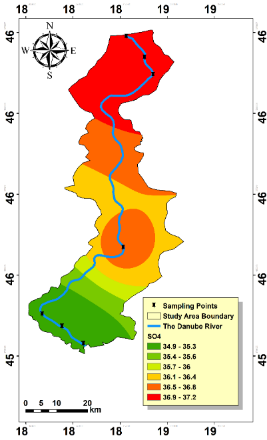  k text here | 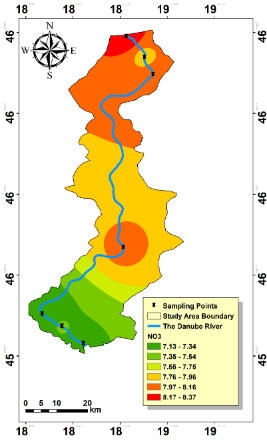 |
| 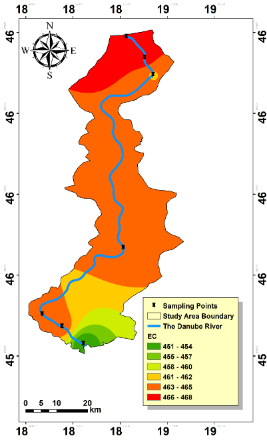  m text here | 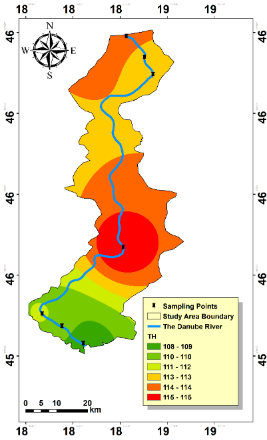  n text here | 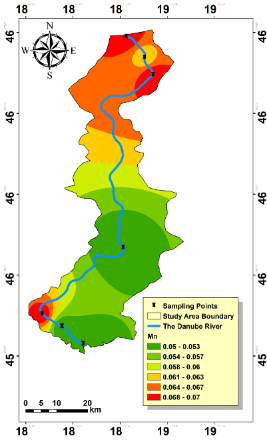  o text here | 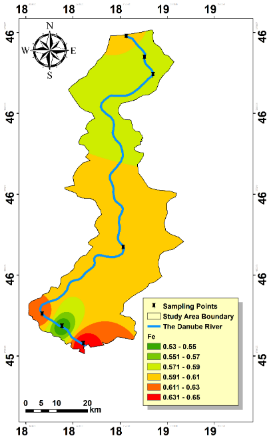  p text here |

Figure 1S Distribution map of the HM and physicochemical parameters: (a) pH, (b)TDS, (c) Temperature, (d) Ca2+, (e) Mg2+, (f) Na+, (g) K+, (h) HCO3−, (i) CO3−, (j) Cl−, (k) SO42−, (l) NO3−, (m) EC, (n) TH, (o) Mn and (p) Fe.

Table 1S Classification of water quality based on WQI values (^1^)

| WQI | Categories | Explanation and Potential Uses |
| --- | --- | --- |
| 0–25 | Excellent | Drinking, Irrigation and Industrial |
| 26–50 | Good | Domestic, Irrigation and Industrial |
| 51–75 | Poor | Irrigation and Industrial |
| 76–100 | Very Poor | Irrigation |
| >100 | Unsuitable for drinking | Restricted use for Irrigation |

Table 2S Statistical analysis and classes of IWQIs.

| Criteria | Min | Max | Mean | Range | Class |
| --- | --- | --- | --- | --- | --- |
| IWQI | 99.64 | 107.66 | 104.36 | 85 - 100 | No restriction |
|  |  |  |  | 70 - 85 | Low restriction |
|  |  |  |  | 55 - 70 | Moderate restriction |
|  |  |  |  | 40 - 55 | High restriction |
|  |  |  |  | 0 - 40 | Severe restriction |
| SAR | 0.37 | 0.68 | 0.49 | <10 | Excellent |
|  |  |  |  | 10–18 | Good |
|  |  |  |  | 19–26 | Fair Poor |
|  |  |  |  | >26 | Unsuitable |
| Na% | 13.75 | 18.72 | 16.11 | <20% | Excellent |
|  |  |  |  | 21%–40% | Good |
|  |  |  |  | 41%–60% | Permissible |
|  |  |  |  | 61%–80% | Doubtful |
|  |  |  |  | > 80% | Unsuitable |
| SSP | 12.52 | 17.59 | 14.88 | <60 | Suitable |
|  |  |  |  | >60 | Unsuitable |
| PS | 0.73 | 1.66 | 1.04 | PS < 3.0 | Excellent to good |
|  |  |  |  | PS = 3.0–5.0 | Good to injurious |
|  |  |  |  | PS > 5.0 | Injurious to unsatisfactory |
| RSC | -1.27 | 0.58 | -0.53 | <1.25 | Good |
|  |  |  |  | 1.25-2.5 | Doubtful |
|  |  |  |  | >2.5 | Unsuitable |

Table 3S Mean values of HQ oral of Mn, Fe and NO3 for adults and children

| Parameters |  | Minimum | Maximum | Mean |
| --- | --- | --- | --- | --- |
|  | Fe | 0.001 | 0.125 | 0.024 |
| HQ Oral for Adult | Mn | 0.025 | 0.439 | 0.074 |
|  | NO_3_^-^ | 0.050 | 0.243 | 0.137 |
|  | Fe | 0.002 | 0.477 | 0.092 |
| HQ Oral for Children | Mn | 0.096 | 1.678 | 0.281 |
|  | NO_3_^-^ | 0.190 | 0.927 | 0.523 |
|  | HIOral for Adult | 0.096 | 0.577 | 0.235 |
| Non-carcinogenic risk | HIOral for Children | 0.366 | 2.201 | 0.896 |

Table 4S Non carcinogenic values of Fe, Mn and NO3 for both Adults and Children

| Samples | Locations | Date | HQ Fe | | HQ Mn | | HQ NO3 | | HI | |
| --- | --- | --- | --- | --- | --- | --- | --- | --- | --- | --- |
|  |  |  | Adults | Children | Adults | Children | Adults | Children | Adults | Children |
| 1 | S1 | 1/9/2019 | 0.12 | 0.48 | 0.13 | 0.48 | 0.18 | 0.70 | 0.43 | 1.65 |
| 2 | S1 | 2/6/2019 | 0.02 | 0.09 | 0.04 | 0.14 | 0.21 | 0.81 | 0.27 | 1.05 |
| 3 | S1 | 3/6/2019 | 0.01 | 0.05 | 0.04 | 0.14 | 0.19 | 0.74 | 0.24 | 0.93 |
| 4 | S1 | 4/3/2019 | 0.02 | 0.07 | 0.19 | 0.72 | 0.18 | 0.70 | 0.39 | 1.48 |
| 5 | S1 | 5/8/2019 | 0.03 | 0.10 | 0.06 | 0.24 | 0.12 | 0.44 | 0.20 | 0.78 |
| 6 | S1 | 6/5/2019 | 0.02 | 0.09 | 0.05 | 0.19 | 0.07 | 0.28 | 0.15 | 0.57 |
| 7 | S1 | 7/3/2019 | 0.01 | 0.04 | 0.05 | 0.19 | 0.08 | 0.29 | 0.14 | 0.52 |
| 8 | S1 | 8/7/2019 | 0.02 | 0.08 | 0.06 | 0.24 | 0.09 | 0.35 | 0.18 | 0.67 |
| 9 | S1 | 9/4/2019 | 0.01 | 0.02 | 0.13 | 0.48 | 0.07 | 0.26 | 0.20 | 0.76 |
| 10 | S1 | 10/2/2019 | 0.01 | 0.03 | 0.19 | 0.72 | 0.23 | 0.87 | 0.42 | 1.62 |
| 11 | S1 | 11/6/2019 | 0.01 | 0.03 | 0.04 | 0.14 | 0.20 | 0.75 | 0.24 | 0.93 |
| 12 | S1 | 12/4/2019 | 0.02 | 0.08 | 0.04 | 0.14 | 0.16 | 0.61 | 0.22 | 0.84 |
| 13 | S2 | 1/9/2019 | 0.10 | 0.39 | 0.13 | 0.48 | 0.18 | 0.70 | 0.41 | 1.56 |
| 14 | S2 | 2/6/2019 | 0.05 | 0.18 | 0.06 | 0.24 | 0.21 | 0.79 | 0.32 | 1.21 |
| 15 | S2 | 3/6/2019 | 0.01 | 0.04 | 0.04 | 0.14 | 0.23 | 0.87 | 0.28 | 1.06 |
| 16 | S2 | 4/3/2019 | 0.02 | 0.07 | 0.06 | 0.24 | 0.16 | 0.63 | 0.24 | 0.93 |
| 17 | S2 | 5/8/2019 | 0.03 | 0.11 | 0.05 | 0.19 | 0.12 | 0.47 | 0.20 | 0.77 |
| 18 | S2 | 6/5/2019 | 0.03 | 0.11 | 0.04 | 0.14 | 0.07 | 0.26 | 0.13 | 0.51 |
| 19 | S2 | 7/3/2019 | 0.01 | 0.05 | 0.13 | 0.48 | 0.07 | 0.28 | 0.21 | 0.81 |
| 20 | S2 | 8/7/2019 | 0.00 | 0.02 | 0.05 | 0.19 | 0.10 | 0.37 | 0.15 | 0.57 |
| 21 | S2 | 9/4/2019 | 0.01 | 0.03 | 0.05 | 0.19 | 0.09 | 0.32 | 0.14 | 0.55 |
| 22 | S2 | 10/2/2019 | 0.01 | 0.04 | 0.06 | 0.24 | 0.20 | 0.76 | 0.27 | 1.04 |
| 23 | S2 | 11/6/2019 | 0.01 | 0.02 | 0.19 | 0.72 | 0.12 | 0.47 | 0.32 | 1.21 |
| 24 | S2 | 12/4/2019 | 0.02 | 0.07 | 0.06 | 0.24 | 0.12 | 0.47 | 0.21 | 0.79 |
| 25 | S3 | 1/9/2019 | 0.10 | 0.39 | 0.13 | 0.48 | 0.21 | 0.79 | 0.43 | 1.66 |
| 26 | S3 | 2/6/2019 | 0.02 | 0.08 | 0.04 | 0.14 | 0.18 | 0.68 | 0.24 | 0.90 |
| 27 | S3 | 3/6/2019 | 0.01 | 0.04 | 0.04 | 0.14 | 0.19 | 0.72 | 0.24 | 0.90 |
| 28 | S3 | 4/3/2019 | 0.02 | 0.07 | 0.06 | 0.24 | 0.20 | 0.76 | 0.28 | 1.08 |
| 29 | S3 | 5/8/2019 | 0.03 | 0.12 | 0.06 | 0.24 | 0.12 | 0.45 | 0.21 | 0.81 |
| 30 | S3 | 6/5/2019 | 0.02 | 0.09 | 0.06 | 0.24 | 0.07 | 0.25 | 0.15 | 0.58 |
| 31 | S3 | 7/3/2019 | 0.01 | 0.04 | 0.05 | 0.19 | 0.07 | 0.26 | 0.13 | 0.49 |
| 32 | S3 | 8/7/2019 | 0.03 | 0.12 | 0.05 | 0.19 | 0.05 | 0.20 | 0.14 | 0.52 |
| 33 | S3 | 9/4/2019 | 0.00 | 0.02 | 0.06 | 0.24 | 0.05 | 0.20 | 0.12 | 0.46 |
| 34 | S3 | 10/2/2019 | 0.01 | 0.02 | 0.25 | 0.96 | 0.21 | 0.82 | 0.47 | 1.80 |
| 35 | S3 | 11/6/2019 | 0.00 | 0.02 | 0.19 | 0.72 | 0.21 | 0.79 | 0.40 | 1.52 |
| 36 | S3 | 12/4/2019 | 0.01 | 0.03 | 0.03 | 0.10 | 0.18 | 0.68 | 0.21 | 0.81 |
| 37 | S4 | 1/9/2019 | 0.11 | 0.42 | 0.13 | 0.48 | 0.17 | 0.66 | 0.41 | 1.56 |
| 38 | S4 | 2/6/2019 | 0.05 | 0.21 | 0.06 | 0.24 | 0.22 | 0.83 | 0.33 | 1.27 |
| 39 | S4 | 3/6/2019 | 0.01 | 0.04 | 0.04 | 0.14 | 0.24 | 0.93 | 0.29 | 1.11 |
| 40 | S4 | 4/3/2019 | 0.02 | 0.06 | 0.05 | 0.19 | 0.21 | 0.81 | 0.28 | 1.06 |
| 41 | S4 | 5/8/2019 | 0.02 | 0.08 | 0.04 | 0.14 | 0.15 | 0.58 | 0.21 | 0.80 |
| 42 | S4 | 6/5/2019 | 0.03 | 0.12 | 0.05 | 0.19 | 0.10 | 0.39 | 0.18 | 0.70 |
| 43 | S4 | 7/3/2019 | 0.01 | 0.03 | 0.13 | 0.48 | 0.05 | 0.19 | 0.18 | 0.70 |
| 44 | S4 | 8/7/2019 | 0.00 | 0.02 | 0.05 | 0.19 | 0.11 | 0.42 | 0.16 | 0.63 |
| 45 | S4 | 9/4/2019 | 0.01 | 0.02 | 0.03 | 0.10 | 0.09 | 0.33 | 0.12 | 0.45 |
| 46 | S4 | 10/2/2019 | 0.01 | 0.04 | 0.04 | 0.14 | 0.10 | 0.39 | 0.15 | 0.57 |
| 47 | S4 | 11/6/2019 | 0.01 | 0.04 | 0.05 | 0.19 | 0.12 | 0.47 | 0.19 | 0.71 |
| 48 | S4 | 12/4/2019 | 0.01 | 0.02 | 0.05 | 0.19 | 0.14 | 0.54 | 0.20 | 0.76 |
| 49 | S5 | 1/10/2019 | 0.10 | 0.38 | 0.13 | 0.48 | 0.20 | 0.75 | 0.42 | 1.61 |
| 50 | S5 | 2/7/2019 | 0.01 | 0.05 | 0.04 | 0.14 | 0.19 | 0.74 | 0.24 | 0.93 |
| 51 | S5 | 3/7/2019 | 0.02 | 0.07 | 0.06 | 0.24 | 0.18 | 0.68 | 0.26 | 0.99 |
| 52 | S5 | 4/4/2019 | 0.02 | 0.06 | 0.06 | 0.24 | 0.20 | 0.76 | 0.28 | 1.06 |
| 53 | S5 | 5/9/2019 | 0.02 | 0.09 | 0.06 | 0.24 | 0.09 | 0.33 | 0.17 | 0.66 |
| 54 | S5 | 6/6/2019 | 0.07 | 0.26 | 0.05 | 0.19 | 0.08 | 0.30 | 0.20 | 0.75 |
| 55 | S5 | 7/4/2019 | 0.01 | 0.03 | 0.04 | 0.14 | 0.05 | 0.19 | 0.10 | 0.37 |
| 56 | S5 | 8/1/2019 | 0.02 | 0.07 | 0.04 | 0.14 | 0.09 | 0.32 | 0.14 | 0.53 |
| 57 | S5 | 9/5/2019 | 0.01 | 0.04 | 0.04 | 0.14 | 0.08 | 0.32 | 0.13 | 0.50 |
| 58 | S5 | 10/3/2019 | 0.01 | 0.04 | 0.06 | 0.24 | 0.12 | 0.45 | 0.19 | 0.73 |
| 59 | S5 | 11/7/2019 | 0.01 | 0.05 | 0.44 | 1.68 | 0.12 | 0.47 | 0.58 | 2.20 |
| 60 | S5 | 12/5/2019 | 0.00 | 0.00 | 0.03 | 0.10 | 0.12 | 0.47 | 0.15 | 0.57 |
| 61 | S6 | 1/10/2019 | 0.02 | 0.09 | 0.04 | 0.14 | 0.18 | 0.70 | 0.25 | 0.94 |
| 62 | S6 | 2/7/2019 | 0.01 | 0.04 | 0.06 | 0.24 | 0.19 | 0.74 | 0.27 | 1.02 |
| 63 | S6 | 3/7/2019 | 0.02 | 0.06 | 0.06 | 0.24 | 0.19 | 0.72 | 0.27 | 1.01 |
| 64 | S6 | 4/4/2019 | 0.03 | 0.12 | 0.06 | 0.24 | 0.10 | 0.38 | 0.19 | 0.73 |
| 65 | S6 | 5/9/2019 | 0.02 | 0.07 | 0.06 | 0.24 | 0.08 | 0.29 | 0.16 | 0.60 |
| 66 | S6 | 6/6/2019 | 0.06 | 0.23 | 0.05 | 0.19 | 0.07 | 0.26 | 0.18 | 0.69 |
| 67 | S6 | 7/4/2019 | 0.02 | 0.07 | 0.04 | 0.14 | 0.05 | 0.20 | 0.11 | 0.41 |
| 68 | S6 | 8/1/2019 | 0.03 | 0.11 | 0.04 | 0.14 | 0.05 | 0.20 | 0.12 | 0.45 |
| 69 | S6 | 9/5/2019 | 0.01 | 0.04 | 0.04 | 0.14 | 0.09 | 0.36 | 0.14 | 0.54 |
| 70 | S6 | 10/3/2019 | 0.01 | 0.05 | 0.25 | 0.96 | 0.20 | 0.76 | 0.46 | 1.77 |
| 71 | S6 | 11/7/2019 | 0.01 | 0.02 | 0.03 | 0.10 | 0.21 | 0.81 | 0.24 | 0.93 |
| 72 | S6 | 12/5/2019 | 0.00 | 0.01 | 0.06 | 0.24 | 0.14 | 0.54 | 0.21 | 0.79 |
| 73 | S7 | 1/10/2019 | 0.12 | 0.45 | 0.13 | 0.48 | 0.19 | 0.74 | 0.44 | 1.67 |
| 74 | S7 | 2/7/2019 | 0.03 | 0.12 | 0.04 | 0.14 | 0.18 | 0.70 | 0.25 | 0.96 |
| 75 | S7 | 3/7/2019 | 0.01 | 0.05 | 0.06 | 0.24 | 0.24 | 0.90 | 0.31 | 1.19 |
| 76 | S7 | 4/4/2019 | 0.02 | 0.06 | 0.05 | 0.19 | 0.20 | 0.78 | 0.27 | 1.03 |
| 77 | S7 | 5/9/2019 | 0.03 | 0.11 | 0.05 | 0.19 | 0.08 | 0.29 | 0.15 | 0.59 |
| 78 | S7 | 6/6/2019 | 0.06 | 0.23 | 0.04 | 0.14 | 0.07 | 0.28 | 0.17 | 0.66 |
| 79 | S7 | 7/4/2019 | 0.01 | 0.02 | 0.13 | 0.48 | 0.05 | 0.20 | 0.19 | 0.71 |
| 80 | S7 | 8/1/2019 | 0.02 | 0.07 | 0.04 | 0.14 | 0.09 | 0.35 | 0.15 | 0.55 |
| 81 | S7 | 9/5/2019 | 0.01 | 0.03 | 0.03 | 0.10 | 0.08 | 0.32 | 0.12 | 0.45 |
| 82 | S7 | 9/12/2019 | 0.01 | 0.03 | 0.04 | 0.14 | 0.09 | 0.33 | 0.13 | 0.51 |
| 83 | S7 | 10/3/2019 | 0.02 | 0.06 | 0.04 | 0.14 | 0.12 | 0.47 | 0.18 | 0.68 |
| 84 | S7 | 11/7/2019 | 0.01 | 0.04 | 0.13 | 0.48 | 0.12 | 0.44 | 0.25 | 0.96 |
| 85 | S7 | 12/5/2019 | 0.01 | 0.03 | 0.03 | 0.10 | 0.16 | 0.61 | 0.19 | 0.74 |
